# From Bile Dysmetabolism to Lipid–Metabolic Remodeling of the Distal Colon: A Vectorial Concept of Colorectal Carcinogenesis (Part I)

**DOI:** 10.3390/cancers18193160

**Published:** 2026-09-30

**Authors:** Maria A. Sukhanova, Zakhar A. Akulov, Zelimkhan G. M. Berikkhanov, Alexey L. Shestakov, Aleksey G. Kotelnikov, Andrey M. Nikolaev, Vadim S. Razumovsky, Evgeniy A. Tarabrin, Milena Yu. Ivanova, Mehrshad Ebrahimnezhad, Sergey Yu. Muraviev

**Affiliations:** 1 Department of Hospital Surgery No. 2, Sechenov First Moscow State Medical University ( Sechenov University), Moscow 119048, Russia; akulov_z_a@student.sechenov.ru (Z.A.A.); berikkhanov_z_g@staff.sechenov.ru (Z.G.M.B.); shestakov_a_l@staff.sechenov.ru (A.L.S.); kotelnikov_a_g@staff.sechenov.ru (A.G.K.); nikolaev_a_m@staff.sechenov.ru (A.M.N.); tarabrin_e_a@staff.sechenov.ru (E.A.T.); ivanova_m_yu_1@staff.sechenov.ru (M.Y.I.); muravev_s_yu@staff.sechenov.ru (S.Y.M.); 2 Cardiology Department No. 2, Yudin City Hospital of Moscow, The Patrice Lumumba Peoples’ Friendship University of Russia (RUDN), Moscow 117198, Russia; mehrshad.ebrahimnejad@gmail.com

**Keywords:** colorectal cancer, colorectal carcinogenesis, bile acids, lipid metabolism, metabolic remodeling, field cancerization, tumor microenvironment, intestinal microbiota, clonal selection, non-neoplastic mucosa

## Abstract

Colorectal cancer (CRC) is traditionally viewed as the result of the sequential accumulation of molecular alterations and the clonal expansion of transformed cells. However, the state of morphologically non-neoplastic mucosa preceding the development of neoplasia remains considerably less well characterized. This narrative review aims to integrate evidence on biliary and lipid metabolism, intestinal transit, microbial metabolism, and field cancerization and to formulate a testable model of early metabolic remodeling in the distal colon. We propose that a chronically altered biliary–metabolic environment, combined with prolonged luminal and mucosa-associated exposure, may promote lipid–metabolic remodeling of morphologically non-neoplastic mucosa. This state is considered not as a new disease entity but as a potential tissue phenotype capable of altering the barrier, metabolic, and regenerative properties of the epithelium. Subsequent exposure to nitrogen-containing products of microbial metabolism may sustain repeated cycles of injury and regeneration, thereby creating conditions for clonal selection. Following the emergence of a neoplastic clone, the same local environment may potentially assume a different biological role and become part of a metabolic niche that supports further clonal expansion. The proposed model extends the concept of field cancerization by incorporating a metabolic dimension and generates a series of spatial and temporal predictions amenable to experimental and prospective clinical testing. Validation of this model may provide a basis for investigating morphologically non-neoplastic mucosa as potential tissue at risk and for developing earlier preventive and diagnostic approaches.

## Simple Summary

Colorectal cancer is usually studied from the point at which abnormal cells and recognizable precancerous lesions have already appeared. However, the surrounding intestinal tissue may begin to change much earlier. In this review, we propose a testable model in which long-term alterations in bile and lipid metabolism, intestinal contents, and transit create a persistent local metabolic environment in the distal colon. We suggest that repeated exposure to this environment may gradually alter the metabolism and function of tissue that still appears non-cancerous, creating conditions that favor further cellular adaptation and selection. This concept does not replace established models of colorectal cancer development but adds a possible earlier metabolic stage. Testing this hypothesis may help researchers identify changes that occur before visible tumors develop and determine whether the surrounding tissue and its metabolic environment could provide new targets for studying cancer risk, early detection, prevention, and disease progression.

## 1. Introduction

Colorectal cancer (CRC) is traditionally viewed as the result of the sequential accumulation of genetic and epigenetic alterations that lead to the emergence and subsequent clonal expansion of neoplastically transformed cells [1]. This model has enabled a relatively detailed reconstruction of the events spanning early molecular alterations to the development of invasive tumors. However, the stage immediately preceding the emergence of an altered cellular clone remains considerably less well defined: what is the state of the tissue before neoplastic transformation begins? The absence of morphological evidence of neoplasia does not necessarily imply the absence of biologically relevant alterations in the surrounding mucosa [2,3].

The concept that apparently unaltered tissue may retain features of an underlying pathological process has been developed within the framework of field cancerization [2]. Originally proposed to explain the occurrence of multiple tumor foci, this concept was subsequently extended to colorectal carcinogenesis. Morphologically unaltered mucosa in patients with CRC has been shown to harbor molecular, genetic, epigenetic, and cellular alterations, some of which vary in magnitude according to their distance from the tumor [2,3,4]. Thus, the boundary between “normal” and neoplastic tissue may be substantially less distinct than suggested by morphological assessment alone.

However, current concepts of field effects predominantly focus on the molecular properties of the tissue [2,3,4]. Considerably less is known about whether a metabolically altered field may precede these changes and create conditions for subsequent injury, regeneration, and selection of altered cellular clones. From this perspective, morphologically non-neoplastic mucosa is no longer merely a nominal “normal control” but becomes an independent object of investigation in early carcinogenesis.

This question is particularly relevant given the biological heterogeneity of CRC along the proximal–distal axis of the colon. Tumors arising in different colonic segments differ in the prevalence of major molecular pathways of carcinogenesis, although current understanding of the spatial heterogeneity of CRC does not support its division into two completely discrete biological categories [5]. The anatomical location of a neoplasm may therefore reflect not only the properties of already transformed cells but also the characteristics of the tissue and luminal environments in which these cells emerge and undergo selection. In this context, the sigmoid colon and rectosigmoid region are of particular interest because of the dynamics of intestinal transit and the potentially prolonged contact of the mucosa with progressively modified luminal contents [6].

Biliary and lipid metabolism may represent one of the factors shaping such an environment. High dietary fat intake, obesity, insulin resistance, and dyslipidemia are associated with CRC risk and may also influence bile acid composition and metabolism [7,8]. In parallel, a growing body of experimental and clinical evidence supports a potential role for secondary bile acids in colorectal epithelial injury and in the regulation of processes associated with carcinogenesis [8]. Thus, systemic metabolic disturbances, diet, biliary metabolism, and the intestinal environment may represent not independent factors but different levels of a common metabolic sequence.

Importantly, the colon is exposed neither to the originally ingested food nor to unmodified bile, but rather to the products of their sequential processing. As intestinal contents progress through the gastrointestinal tract, nutrients and water are absorbed, bile acids are reabsorbed, residual substrates undergo microbial transformation, and the physicochemical properties of the luminal contents change [8]. The effect on the mucosa is therefore determined not only by the presence of an individual potentially harmful compound but also by its concentration, duration of contact, local chemical environment, and rate of clearance. Under conditions of slow transit, exposure time itself may become a key factor that converts a physiologically tolerable residual metabolic load into chronic local exposure [6].

This perspective provides a different framework for considering the relationship between biliary metabolism and CRC. A substantial body of research has focused on individual bile acids, microbial metabolites, dietary factors, obesity, or the metabolism of established tumors [8,9]. Far less attention has been paid to what occurs within the intestinal wall during the interval between prolonged exposure to an altered luminal environment and the emergence of morphologically detectable neoplasia. This interval may encompass an early tissue phase of disease that remains undetected within the conventional sequence of “risk factor–mutation–tumor”.

In the present review, we consider the possibility that such an intermediate state exists. Our central hypothesis is that a chronically altered biliary–metabolic environment may induce persistent changes in the metabolic and morphofunctional state of the distal colonic wall before overt neoplastic transformation occurs. Within this framework, the proposed concept does not oppose field cancerization but rather extends it: the established molecular alterations of morphologically non-neoplastic mucosa may potentially be preceded by an earlier phase of local metabolic remodeling.

The aim of this narrative review is to analyze the relationship between biliary–lipid exposure, intestinal transit, and the state of morphologically non-neoplastic mucosa in the distal colon and, on this basis, to formulate a testable model of an early tissue phase of colorectal carcinogenesis. The central question precedes the conventional starting point of tumor biology: what occurs in the intestinal wall before a cell emerges that can already be recognized as neoplastic? If such a preceding tissue phase indeed exists, it may represent the link between chronic metabolic exposure and the initiation of neoplastic evolution, while also providing a potential window for earlier detection of the pathological process. However, the implications of this concept are not limited to prevention and early diagnosis. Understanding the sequence of changes that shapes the tissue environment before tumor development and continues to operate after its emergence may potentially alter the framework through which therapeutic effects are evaluated. In this context, surgical tumor removal, chemotherapy, or targeted therapy may be considered not only in terms of their effects on an established neoplastic clone but also in terms of whether the local metabolic conditions that preceded its emergence and may support subsequent clonal expansion persist after treatment.

This perspective may have implications across the continuum of clinical decision-making, from the selection of systemic anticancer therapy and assessment of recurrence risk to the extent of radical intervention and the rationale for palliative treatment. This does not imply a ready-to-use algorithm for modifying current therapeutic approaches, but rather a broader principle: if a tumor develops within a chronically altered tissue and metabolic field, future therapeutic strategies may need to consider not only the properties of the tumor itself but also the state of the environment in which it arose and continues to exist. Accordingly, modifying or eliminating conditions that support the selective advantage of tumor cells could potentially be investigated as an additional means of influencing the subsequent disease trajectory, including progression of existing metastatic lesions, development of new neoplastic lesions, and the likelihood of recurrence after treatment.

Thus, investigation of the pre-neoplastic metabolic state of the intestinal wall may be relevant not only to the identification of earlier markers of CRC but also to the development of a more integrated understanding of disease prevention, treatment, and long-term control.

## 2. Materials and Methods

This study was conducted as a narrative literature review with a conceptual synthesis of the available experimental, clinical, and pathophysiological evidence. The aim was not to quantitatively pool study results but to assess, in a structured manner, the biological plausibility of individual components of the proposed model linking biliary–lipid metabolism, intestinal transit, the luminal and mucosa-associated environments, metabolic remodeling of morphologically non-neoplastic mucosa, and the early stages of colorectal carcinogenesis.

The literature search was conducted in PubMed/MEDLINE, Scopus, and Web of Science. The most recent structured search was performed on 1 July 2026. The thematic search domains, representative search terms and their combinations, and the approach to literature selection are presented in Supplementary Table S1. Reference lists of relevant original studies and review articles were additionally screened to identify publications not retrieved through the initial search. No date restrictions were applied, allowing historically important experimental studies to be integrated with contemporary high-quality evidence. The principal criteria for source selection were methodological rigor and direct relevance to the mechanisms under consideration; accordingly, preference was given to peer-reviewed original studies and systematic reviews that most directly addressed these mechanisms and had adequate methodological support.

The search was organized around several interconnected thematic domains: enterohepatic circulation and bile acid pool composition; secondary bile acids and colorectal carcinogenesis; dietary and tissue lipid metabolism; intestinal transit and the duration of contact between luminal contents and the mucosa; microbial transformation of luminal substrates; ammonia, ammonium, and other products of proteolytic metabolism; field cancerization and alterations in morphologically non-neoplastic mucosa; regeneration and phenotypic plasticity of the intestinal epithelium; spatial molecular heterogeneity of CRC; and metabolic reprogramming and clonal evolution of tumor cells.

The review primarily included original experimental and clinical studies directly characterizing individual mechanisms relevant to the proposed model. Contemporary systematic and narrative reviews were used to synthesize broader biological domains and to identify primary studies. When several potentially relevant sources were available, preference was given to more recent publications, original studies, and studies directly addressing colorectal tissue or CRC. Earlier publications were retained when they represented historically important or foundational experimental concepts for which no equivalent contemporary primary study was available.

Studies of morphologically non-neoplastic mucosa in patients with colorectal neoplasia were analyzed separately. Molecular, genetic, epigenetic, transcriptomic, and metabolic alterations identified in macroscopically or histologically unaltered tissue were considered, together with their spatial relationship to the distance from the tumor. These data were used to compare the proposed metabolic remodeling with established concepts of field cancerization and field defects.

For the biliary–metabolic component, we separately evaluated evidence on enterohepatic circulation, microbial transformation of bile acids, the effects of diet and systemic metabolic status on bile acid composition, and experimental evidence regarding their effects on epithelial barrier integrity, oxidative stress, DNA damage, and inflammatory and proliferative signaling pathways. For the lipid component, we considered mechanisms of intestinal lipid transport, intracellular lipid storage and redistribution, lipid droplet formation, and alterations in membrane organization. For the nitrogen-containing component, we analyzed microbial production of ammonia and other products of proteolytic metabolism, the pH dependence of the ammonia–ammonium system, and evidence regarding their potential effects on the colorectal epithelium.

Because the proposed model places particular emphasis on the distal colon, we additionally analyzed studies addressing proximal–distal heterogeneity in CRC and regional differences in intestinal transit and microbial fermentation. Distal localization was not regarded a priori as the site of maximal exposure; rather, these data were used to assess biological plausibility and to formulate spatially testable predictions of the model.

The evidence was synthesized from factors determining the composition of the luminal environment to potential alterations in the intestinal wall and subsequently to conditions capable of influencing injury, regeneration, clonal selection, and the metabolic properties of established neoplasia. On this basis, five interconnected domains of analysis were defined: chronic biliary–metabolic exposure; potential lipid–metabolic remodeling of the morphologically non-neoplastic intestinal wall; exposure of previously altered epithelium to a nitrogen-containing metabolic environment; spatial heterogeneity of metabolic exposure and its potential relationship to clonal expansion; and the formation of a metabolic niche following the emergence of neoplasia.

A key methodological principle was to distinguish evidence directly supported by existing studies from the authors’ conceptual integration of that evidence. Enterohepatic circulation, microbial transformation of bile acids, dietary lipid metabolism, the production of nitrogen-containing microbial metabolites, the molecular effects of secondary bile acids, field cancerization, and metabolic reprogramming of tumor cells were regarded as the established evidence base. In contrast, the proposed temporal sequence—“altered biliary–luminal environment → lipid–metabolic remodeling of morphologically non-neoplastic mucosa → additional nitrogen-containing exposure → repeated injury and regeneration → clonal selection”—represents an author-generated, testable hypothesis and is not presented as an established mechanism of colorectal carcinogenesis.

Given the heterogeneity of the evidence supporting individual components of the model, four levels of empirical support were additionally distinguished: human evidence directly related to colorectal tissue or CRC; evidence from experimental animal and in vitro studies; indirect evidence derived from other tissues or biological models; and author-proposed hypothetical links for which direct evidence is currently lacking. This distinction was not intended as a formal system for grading the quality of evidence, but rather as a means of indicating the degree of direct empirical support for individual elements of the concept. A summary of the evidence base is presented in Table 1.

The terms “lipid–metabolic remodeling”, “steatotic component of remodeling”, and “reactive plateau” are used in this review as operational terms for the proposed biological states. They are not established diagnostic categories and do not imply the existence of a distinct nosological entity of colonic steatosis or metabolic disease of the colon. Similarly, the proposed relationships between the spatial distribution of metabolites, the direction of clonal expansion, and subsequent tumor growth are considered hypotheses that require direct experimental and prospective clinical validation.

## 3. The Sigmoid Colon as a Potential Site of Chronic Biliary–Metabolic Exposure

The relationship between bile acids and colorectal carcinogenesis has been investigated for several decades. Particular attention has been given to secondary bile acids, especially deoxycholic and lithocholic acids, which at elevated concentrations can impair epithelial barrier function, induce oxidative stress and DNA damage, and activate inflammatory and proliferative signaling pathways. Experimental evidence indicates that prolonged increases in intestinal exposure to certain secondary bile acids can promote the development of colorectal neoplasia [8,11,14]. Thus, the potential carcinogenic effects of an altered bile acid pool are not in themselves a novel concept. A less clearly defined question is how chronically altered biliary metabolism may reshape the broader environment to which the distal mucosa is exposed before a tumor develops.

Under physiological conditions, most bile acids return to the enterohepatic circulation following reabsorption in the terminal ileum. However, this process is not complete. A proportion of bile acids reaches the colon, where they undergo bacterial deconjugation and further transformation [14,15]. The colon is therefore continuously exposed to biliary derivatives, and their mere presence cannot be regarded as pathological. Rather, their quantity, composition, and duration of contact with the mucosa are likely to be relevant.

This distinction is fundamental to the proposed model. We focus not on the presence of bile in the colon per se, but on a disturbance in the balance among the delivery of biliary-dependent substrates, their transformation, absorption, and subsequent elimination. High-fat diets, obesity, and associated systemic metabolic disturbances can alter bile acid synthesis and pool composition, whereas changes in the intestinal microbiota influence their subsequent transformation [7,10,14]. Consequently, the intestinal epithelium is exposed not to a single isolated compound but to a dynamically changing environment comprising primary and secondary bile acids, residual dietary components, and products of their microbial metabolism.

In this context, we propose a distinction between the biliary factor and biliary–metabolic exposure. The former can be assessed through the concentrations of individual bile acids and other bile constituents. The latter refers to the resulting chemical environment that reaches a particular intestinal segment after successive stages of digestion, reabsorption, and microbial transformation [14,15]. However, local exposure depends not only on which substances reach a given segment but also on what happens to them thereafter. When intestinal contents are retained, the dietary load ceases to represent merely a transient external exposure and begins to shape the dynamics of the local environment: substrate concentrations, the relative abundance of transformation products, and the rate at which the system returns to its baseline state all change. Thus, the pattern of food intake—the amount, composition, and frequency of substrate delivery—may determine the rhythm and rate of recurrent fluctuations in the chemical composition of the luminal contents, producing a state of metabolic instability rather than a sustained increase in the concentration of one or several agents. This distinction is important for the intestinal epithelium, which is exposed not to isolated deoxycholic acid or a single dietary lipid fraction but to a complex mixture of compounds whose properties continue to change within the intestinal lumen.

As intestinal contents progress through the colon, water is absorbed, fecal material becomes more concentrated, and substrate availability for microbial metabolism changes. At the same time, the residence time of the contents becomes an independent variable. The same amount of a potentially harmful substance therefore does not necessarily impose the same tissue burden in different intestinal segments. Brief exposure to a relatively high concentration and prolonged contact with a lower concentration represent biologically distinct patterns of exposure.

This is particularly relevant to the sigmoid colon and the rectosigmoid junction. We do not propose a fixed anatomical boundary beyond which intestinal contents suddenly acquire carcinogenic properties. Rather, chemical and functional changes develop progressively along the colonic axis. Within the proposed framework, the sigmoid colon and rectosigmoid junction are considered potentially relevant sites for testing the hypothesis of chronic local exposure, because concentration of intestinal contents may coincide with relatively prolonged mucosal contact and, when motility is reduced, additional retention in these regions [6]. This does not imply that maximal biliary–metabolic exposure necessarily occurs in these segments; its segmental distribution must be established through direct studies of the luminal and mucosa-associated environments. If such an environment persists over time, its effects may involve several colonic regions, resulting in a spatially heterogeneous pattern of exposure. Reduced motility may further prolong local contact, increasing not only the duration of direct interaction between luminal contents and the mucosa but also the time available for further microbial, epithelial, and chemical transformation of residual substrates.

Stasis of intestinal contents should therefore not be regarded merely as a mechanical prolongation of exposure. The luminal contents continue to change during their residence in the colon. Microorganisms transform bile acids and lipid- and nitrogen-containing compounds; local changes in acidity influence the properties of the resulting metabolites; and the epithelium simultaneously absorbs and exchanges substances with the mucosa-associated environment [14,30]. These processes are not mediated by bacteria alone: the local biochemical system reflects the combined activity of the microbial community, epithelial cells, and reactions among compounds that have already been generated. Thus, slower transit alters not only the duration of contact but also the conditions under which individual transformations continue and, consequently, potentially the composition of substances present immediately adjacent to the mucosal surface [6,30].

The near-mucosal layer is of particular importance in this model. It is here that the chemical contents of the lumen directly interact with the mucosal surface and that microbial transformation, diffusion, absorption, and intermolecular reactions occur in close proximity to epithelial cells [31,32]. Consequently, the average composition of the intestinal contents and the actual tissue exposure are not necessarily equivalent: the local mucosa-associated environment may differ substantially from the more central portions of the luminal contents. The combination of local microbial activity, tissue processes, diffusion, and intermolecular transformations may generate a chemical environment that directly determines how luminal contents interact with epithelial cells. In this sense, the mucosa-associated environment, rather than the averaged luminal environment alone, is of particular interest for testing the proposed model.

In this regard, the long-recognized relationship among intestinal transit, luminal acidity, and products of nitrogen metabolism is particularly relevant. It has previously been proposed that prolonged residence time and altered acidity may increase mucosal exposure to free ammonia, whereas greater intestinal content volume and accelerated transit may reduce such exposure [19]. Subsequently, microbial metabolites and the altered luminal environment came to be considered among the factors potentially involved in colorectal carcinogenesis [33]. Thus, the concept that the duration of mucosal contact with chemically altered intestinal contents may have carcinogenic relevance is supported by the literature. A more detailed analysis of the mechanisms that maintain or disrupt these dynamics is beyond the scope of the present study and will be addressed separately in subsequent publications in this series; the key point here is that the intestinal environment is a continuously renewed and chemically evolving system rather than a static collection of metabolites. Such dynamics may be particularly pronounced with diets characterized by high fat and carbohydrate loads and with regular consumption of foods subjected to intensive thermal processing, including frying and grilling [11,34]. Under these conditions, not only the amount of incoming substrates but also their chemical structure changes, potentially broadening the range of subsequent intraluminal and mucosa-associated transformations and thereby modifying the nature of metabolic exposure at the mucosal surface [34].

The proposed model takes this concept a step further. We suggest that not only the direct damaging effects of individual components of the luminal contents but also the response of the intestinal wall to chronically recurrent exposure may be relevant. Each new digestive cycle generates a renewed influx of biliary and dietary substrates. When their processing is persistently impaired, the distal mucosa may repeatedly encounter similar, although not identical, metabolic loads. Within the proposed model, the potentially relevant unit of exposure is therefore not merely an individual episode of contact but a recurring sequence of “delivery–transformation–retention–mucosal interaction–partial elimination–renewed delivery”.

It is the recurrence of this sequence that distinguishes the proposed mechanism from the classical model of exposure to a single exogenous carcinogen. For external carcinogenic factors, the conventional framework involves repeated exposure to one or more damaging compounds. In the proposed model, the potential disruption of intrinsic mechanisms that normally maintain the relative stability of intestinal content composition becomes central. The potentially relevant factor is therefore not a single molecule but dynamic metabolic instability: a recurrent shift in the chemical environment after each dietary exposure, with incomplete restoration of physiological equilibrium before the next influx of substrates.

From this perspective, the biliary component is important not because it represents the sole source of potentially carcinogenic compounds, but because it contributes to maintaining one of the fundamental properties of the digestive environment—the processing, solubilization, redistribution, and subsequent fate of lipid substrates [14,15]. Disruption of this regulation may render the consequences of an otherwise ordinary dietary load substantially more variable and dependent on dietary composition and intake patterns.

This framework also provides a different perspective on the relationship between systemic metabolism and local carcinogenesis. Obesity, insulin resistance, dyslipidemia, and dietary factors may influence CRC risk through multiple pathways [7,8]. Within the proposed model, they are also relevant as potential modifiers of the initial biliary–lipid load that is subsequently processed within the intestine [10,14]. Thus, the systemic metabolic phenotype and the local colonic environment need not represent competing explanations of risk; within the proposed framework, they are considered potentially interconnected levels of the same pathophysiological process.

It is equally important to distinguish cause from consequence. Slower transit may prolong chemical exposure, but injury to the intestinal wall may itself impair motility. As the process evolves, these relationships may become bidirectional: an initial slowing of transit increases exposure time, whereas subsequent alterations in intestinal wall function may further sustain stasis. The model therefore allows for the formation not only of a linear sequence but also of a self-sustaining pathophysiological loop.

From a research perspective, this shifts the object of observation. If chronic exposure can indeed precede morphologically evident neoplasia, the relevant object is not limited to an already detected tumor or polyp but also includes morphologically non-neoplastic mucosa chronically exposed to an altered luminal and mucosa-associated metabolic environment. Studying this environment may provide additional information on the local conditions governing chemical exposure of epithelial cells that cannot be obtained from the averaged composition of intestinal contents alone.

Thus, within the proposed model, the formation of a zone of chronic exposure is determined by at least three interconnected parameters. The first is the qualitative and quantitative composition of substances reaching a particular intestinal segment. The second is the nature of the reactions among these substances, the microbial community, the epithelium, and other components of the mucosa-associated environment—that is, what the original substrate becomes within the intestine. The third is process kinetics: the rates at which new substances enter the system, undergo chemical and biological transformation, are absorbed, and are ultimately eliminated with the intestinal contents. It is the combination of composition, reaction dynamics, and temporal kinetics, rather than the concentration of a single potentially damaging agent, that defines the local metabolic exposure considered in this model.

For the conceptual description of a state arising from a relative balance between the continuous arrival of chemically active luminal contents and their delayed clearance from a particular intestinal segment, we propose the working term “reactive plateau”. Its formation can be viewed as a dynamic process: luminal contents initially reach a given segment with an established set of ongoing chemical and microbial reactions; when peristalsis is slowed, their local residence time increases while intraluminal and mucosa-associated transformations continue. As a result, a chemically active environment may persist within a given region for a period of time, with the intensity of exposure determined not only by the concentrations of individual compounds but also by the rates of their formation, transformation, absorption, and elimination. Within this framework, such zones may occur in several regions of the colon and generate a spatially heterogeneous pattern of metabolic exposure; the term “reactive plateau” is a descriptive construct rather than an established physiological or pathological category.

The presence of such a state does not, in itself, imply inevitable tissue injury. Its biological outcome depends on the balance between two dynamic variables: the intensity and duration of chemical exposure, on the one hand, and epithelial resistance and the efficiency of barrier, metabolic, and regenerative mechanisms, on the other. As long as the compensatory capacity of the mucosa is sufficient to meet the imposed load, tissue homeostasis may be maintained. However, if the intensity and duration of local exposure exceed the compensatory capacity of the epithelium, such an environment is considered within the proposed model to represent a potential condition for persistent injury and subsequent remodeling of the intestinal wall.

At this point, the biliary hypothesis extends to a further level. Bile acids can directly affect the epithelium, but bile also determines the efficiency of dietary lipid digestion and absorption [8,14,15]. Alterations in the biliary environment may therefore modify not only the spectrum of bile acids reaching the colon but also the fate of the lipid substrate itself. If a proportion of this load consistently reaches the distal colon, the next question concerns the tissue rather than the luminal contents: can recurrent residual lipid exposure leave a persistent metabolic imprint on the colonic wall, and might such an effect be particularly pronounced in its distal segments?

## 4. From Residual Lipid Load to Lipid–Metabolic Remodeling of the Colonic Wall

If the preceding stage of the model describes the development of prolonged biliary–metabolic exposure, the next fundamental question concerns not the composition of the intestinal contents but the response of the tissue itself. For the proposed concept, it is not sufficient to demonstrate that an altered bile acid pool or an increased amount of residual lipids reaches the colon. It is necessary to determine whether recurrent lipid exposure can leave a persistent metabolic imprint on the morphologically non-neoplastic intestinal wall.

The relationship between lipid metabolism and CRC is well established at several levels. High-fat diets, obesity, dyslipidemia, and insulin resistance are associated with an increased risk of CRC, whereas tumor cells exhibit pronounced reprogramming of lipid synthesis, uptake, storage, and oxidation [7,9,11]. At the same time, high dietary fat intake alters bile acid secretion and composition, while the intestinal microbiota determines their subsequent transformation. Thus, the distinction among dietary, systemic, biliary, and tissue lipid metabolism is to a considerable extent conditional: these processes represent interconnected levels of a metabolic system, different components of which may simultaneously influence the state of the colorectal epithelium.

Within this system, the effects of secondary bile acids have been studied most extensively. Deoxycholic and lithocholic acids can affect cellular membranes, redox balance, and inflammatory and proliferative signaling pathways [8,30]. Experimental studies have shown that deoxycholic acid can promote processes associated with DNA damage, activation of Wnt/β-catenin signaling, increased cyclooxygenase-2 expression, altered bile acid receptor activity, and remodeling of the antitumor immune response [8,12,13]. In experimental models, prolonged elevation of intestinal exposure to deoxycholic acid has been associated with the development of colorectal neoplasia [8,12]. These observations provide a strong basis for linking altered biliary metabolism to carcinogenesis, although they predominantly describe the effects of individual bile acids.

The proposed model shifts the focus toward another potential consequence of biliary disturbances. Bile is important not only as a source of biologically active bile acids but also as an essential component of dietary lipid digestion and absorption [14,15]. Alterations in bile composition and the efficiency of micelle formation may therefore modify the fate of the lipid substrate itself [15]. This load may become particularly pronounced with regular consumption of large amounts of fat and foods subjected to intensive thermal processing, which results in the formation of additional chemically modified dietary components, including advanced glycation end products [11,34]. In this context, not only the absolute amount of fat but also the physicochemical form of lipid compounds undergoing bile-mediated digestion may also be relevant. If processing and absorption are incomplete, a proportion of lipid components may remain within the intestinal contents and reach more distal segments. The colonic mucosa may therefore be simultaneously exposed to biliary derivatives and a residual lipid load.

However, the presence of unabsorbed lipids in the lumen does not imply their accumulation within the intestinal wall. This distinction is fundamental. At present, there is insufficient evidence to conclude that chronic biliary–lipid exposure induces a state in the sigmoid colon directly analogous to hepatic steatosis. Nevertheless, the analogy with steatosis may be conceptually useful in a limited sense—as a model of mismatch between lipid substrate delivery and the capacity of the tissue to process, store, and eliminate it [18]. It does not imply identical mechanisms or morphological manifestations in the liver and intestinal wall. Accordingly, the central focus of the proposed hypothesis is not “colonic steatosis” as a new disease entity, but lipid–metabolic remodeling—a potential alteration in lipid distribution, storage, and utilization within the intestinal wall under conditions of prolonged atypical exposure. Direct evidence demonstrating that chronic luminal lipid exposure produces persistent pre-neoplastic remodeling of morphologically unaltered human colonic mucosa remains limited; this transition therefore represents one of the central testable components of the proposed model.

It is essential to distinguish several interconnected levels: the presence and composition of lipids in the intestinal lumen, their uptake by epithelial cells, their intracellular processing and storage, and, ultimately, remodeling of lipid metabolism at the tissue level. The first three processes provide biological prerequisites for the last but are not equivalent to it. If lipid overload becomes a tissue-level phenomenon, substrate redistribution may involve several compartments of the intestinal wall, including epithelial cells, the lamina propria and submucosa, tissue macrophages and other immune cells, and, with deeper involvement, smooth muscle elements. In this context, lipid–metabolic remodeling represents not merely intracellular lipid accumulation but a potential reorganization of lipid distribution across different cellular and tissue compartments. It is the involvement of multiple compartments and the associated lipid-related tissue response that distinguishes the proposed remodeling both from the presence of lipids within the lumen and from their transient intracellular accumulation.

The biological plausibility of such a transition is supported by the fact that the intestinal epithelium is not a passive barrier. Lipids contribute to cell membrane formation, energy metabolism, and intracellular signaling [35]. Cholesterol and sphingolipids determine the organization of membrane microdomains that influence receptor localization and the activity of multiple signaling pathways [16]. Fatty acids may be incorporated into structural lipids, oxidized, esterified, or stored in intracellular lipid droplets [35]. Altering the balance among lipid uptake, utilization, storage, and elimination may therefore theoretically modify epithelial cell properties even before the emergence of a neoplastic phenotype.

Intracellular lipid droplets are of particular interest in this context. Their formation should not automatically be interpreted as evidence of injury. On the contrary, transient storage of fatty acids in neutral form may serve a protective function by limiting the lipotoxic effects of free lipid species [17]. However, when cellular processing capacity is chronically exceeded, an adaptive mechanism may potentially develop into a persistent phenotype. Thus, the proposed accumulation of lipids in epithelial cells should primarily be regarded as a potential marker of altered tissue homeostasis rather than evidence that carcinogenesis has already begun. Moreover, the presence of lipid droplets in individual epithelial cells does not itself constitute tissue remodeling; the latter implies a more persistent alteration in lipid homeostasis involving multiple cellular and tissue compartments.

The functional shift within the cell itself is particularly important. Colonic epithelial cells are physiologically specialized primarily for maintaining barrier function, water and electrolyte transport, and interactions with the local luminal environment. Under conditions of chronic atypical lipid loading, lipid uptake, intracellular storage, and processing—processes that are not dominant functions of the distal colorectal epithelium—may assume greater importance. In this context, metabolic remodeling should be understood not only as substrate accumulation but also as a potential alteration in the metabolic profile and functional state of the cell.

It is in this restricted sense that we use the term “steatotic component of remodeling”. It denotes the proposed pathological redistribution and accumulation of lipids within the intestinal wall and does not imply direct extrapolation of the mechanisms of steatotic liver disease to the colon. However, the absence of an established concept of “steatotic injury” of the colon leaves open the possibility of a tissue phenotype that is functionally analogous but morphologically distinct. Such changes may have remained relatively understudied because intestinal lipid metabolism has traditionally been considered primarily in the context of absorption and transport rather than chronic remodeling of lipid homeostasis within the intestinal wall itself.

Additional support for considering lipid-associated alterations as a potential tissue-level rather than exclusively epithelial phenomenon comes from individual morphological observations in humans: aggregates of lipid-laden macrophages have been described in the lamina propria adjacent to distal colorectal neoplasms, as have rare lipid accumulations involving multiple cell types within the sigmoid colonic mucosa. These observations do not establish a pre-neoplastic nature of the proposed remodeling but demonstrate the possibility that lipid-associated alterations may extend beyond a single epithelial compartment.

This issue is particularly important in CRC research, where morphologically unaltered mucosa is frequently used as control tissue for comparison with tumors. The absence of dysplasia or tumor cells does not guarantee that such mucosa is metabolically equivalent to tissue from healthy individuals [2,3,4]. Evidence on field cancerization has already demonstrated that macroscopically and histologically relatively preserved mucosa may harbor molecular and epigenetic alterations. The proposed model extends this concept by suggesting that disruption of local lipid homeostasis may represent one component of such an altered tissue field, potentially involving not only the epithelium but also other compartments of the intestinal wall.

Reverse causality must be considered in this context. Detection of increased lipid content in mucosa adjacent to a tumor would not, by itself, establish that lipid remodeling preceded cancer development. Tumors can substantially alter the metabolism of surrounding tissues, blood supply, the inflammatory environment, and substrate availability. It is therefore particularly important to search for a similar lipid phenotype in tissues that do not yet contain a neoplastic lesion and to assess the spatial distribution of these alterations across different colonic segments, including the sigmoid colon and rectosigmoid junction. For the proposed model, the critical issue is not simply the presence of lipid accumulation adjacent to a tumor, but whether such alterations can exist independently of an established tumor microenvironment and potentially before morphologically detectable neoplasia.

If the proposed lipid remodeling exists before the emergence of neoplasia, its consequences may extend well beyond simple lipid accumulation. Alterations in membrane composition may affect barrier function and intercellular contacts, while an excess of specific lipid species may sustain oxidative and inflammatory responses [16,17]. In this context, inflammation may become one component of a lipid-associated tissue response, linking altered substrate metabolism to broader remodeling of the local microenvironment. The potential involvement of monocytes and macrophages is of particular interest, as these cells can respond to changes in the lipid environment and may thereby connect metabolic overload with the immune component of the tissue response. Initially, such a response may contribute to restoration of local homeostasis, removal of damaged cells, and return of the tissue to its baseline state. However, with continuous lipid exposure, the underlying stimulus is not removed, and the compensatory inflammatory response may become chronic.

Under these conditions, epithelial injury may potentially be accompanied by local edema, altered vascular patterns, disruption of mucosal architecture, mucus hypersecretion, and altered regeneration. If such changes indeed accompany the proposed remodeling, morphologically non-neoplastic tissue may no longer remain functionally equivalent to its baseline state despite the absence of neoplastic growth. Clinically, such a state could theoretically overlap with features of left-sided colitis or sigmoiditis, including pain or discomfort in the left abdomen, bloating, mucus discharge, and variable bowel habits. Of particular interest within the proposed concept is the alternation between episodes of stool retention and diarrhea. This pattern need not necessarily reflect alternating hypo- and hypermotility: if slowed transit remains the predominant motor disturbance, loose stools could theoretically be related to impaired water reabsorption and altered barrier function of the affected epithelium. This interpretation requires confirmation and does not justify the use of these symptoms as specific indicators of lipid remodeling; at present, they should therefore be regarded only as potential clinical correlates to be evaluated alongside objective tissue characteristics.

Similarly, excessive mucus production may be of interest as a possible but nonspecific correlate of a local tissue response. Under conditions of chronic injury, increased secretory activity of the mucosal glandular apparatus may represent another protective response aimed at separating the epithelium from an unfavorable mucosa-associated environment.

Any subsequent alteration in cellular differentiation requires particularly cautious interpretation. Chronic exposure to a metabolic environment atypical for a given intestinal segment could theoretically create conditions favoring the persistence of cellular phenotypes better adapted to that environment. The specific hypothesis of small-intestinal-type metaplasia in the sigmoid colon currently requires direct morphological and molecular confirmation. At this stage, it is therefore more appropriate to refer to metabolically driven phenotypic remodeling of the mucosa without prespecifying a particular morphological subtype.

Nevertheless, the possibility of metaplastic adaptation is not biologically exceptional. In other organs, chronic exposure to a chemical or mechanical environment atypical for a given tissue can lead to replacement of the original cellular phenotype by one that is more resistant to the new conditions. Prolonged exposure of the colon to an increased lipid load could therefore theoretically be accompanied by alterations in the morphofunctional specialization of the epithelium. In this context, the key issue is not a specific histological type of metaplasia but the proposed relationship among environmental alteration, functional overload, metabolic adaptation, and persistent changes in cellular phenotype. These processes are presented sequentially in the model for analytical clarity, although biologically they may partially overlap and influence one another.

The research significance of this hypothesis lies in the possibility of identifying a potentially metabolically altered tissue state before a polyp or dysplasia develops. If chronic biliary–lipid exposure indeed leaves a persistent tissue imprint, it should be investigated simultaneously at several levels: intracellular lipid distribution, lipid composition of biopsy specimens, the state of the epithelium, submucosa, immune and smooth muscle cells, membrane characteristics, and mucosal morphology. Comparison of these parameters with the composition of intestinal contents and the anatomical location of the sampled region is particularly important. Such a design would allow simultaneous testing of both the existence of the proposed remodeling and its potential segmental predominance.

From a clinical perspective, areas of nonspecifically altered distal mucosa—showing edema, mucosal flattening, mucus hypersecretion, or features of chronic irritation—may be of particular interest for further metabolic and morphological investigation. These features are nonspecific and cannot themselves be used to diagnose lipid–metabolic remodeling. However, comparing them with tissue lipid distribution, the state of the immune compartment, and characteristics of the luminal environment may help determine whether the proposed metabolic pattern of tissue alteration exists.

Thus, the key transition in the proposed model lies neither between “fatty food” and cancer nor between an individual bile acid and a mutation. Rather, it lies between a chronically altered luminal and mucosa-associated environment and a potential persistent alteration in tissue homeostasis. This alteration may involve not a single epithelial cell but the intestinal wall as a functionally interconnected system comprising the epithelium, immune cells, the submucosal compartment, and the motor apparatus. In this context, lipid–metabolic remodeling represents not simple fat accumulation but a proposed lipid-associated tissue response capable of modifying how the tissue subsequently responds to ongoing chemical exposure.

Lipid remodeling alone, however, does not explain the transition to mutagenesis. On the contrary, it may initially form part of an adaptive response that enables the tissue to persist under conditions of chronic overload. Its potential relevance to carcinogenesis may emerge through interactions with other components of the continuously changing luminal environment, including products of nitrogen metabolism. In the analytical structure of the model, this component is considered next; however, this ordering does not imply an obligatory strict chronology, as lipid adaptation, nitrogen-containing exposure, and epithelial injury may partially overlap in time and modify one another’s effects.

## 5. Nitrogen-Containing Metabolic Environment and Injury to Metabolically Altered Epithelium

If lipid–metabolic remodeling represents a potential first persistent tissue response to chronically altered biliary–luminal exposure, it does not by itself explain the transition from adaptation to neoplastic evolution. The term “evolution” is particularly important in this context. A mutation does not arise with the purpose of driving a cell toward cancer; rather, the cellular population changes progressively under sustained selective pressure [20]. If metabolic instability of the environment becomes an almost persistent condition for the tissue, cellular remodeling may reflect not only dysregulation but also a transition toward a new morphofunctional state better adapted to survival under these conditions. In this restricted, selection-based sense, the subsequent development of a neoplastic phenotype can indeed be considered a form of cellular evolution. Moreover, the initial redistribution of lipids may serve predominantly as a compensatory mechanism, allowing epithelial cells to limit lipotoxic effects and maintain cellular homeostasis [17]. Further progression may require an additional exposure capable of sustaining injury within the altered tissue. One candidate is the nitrogen-containing metabolic environment that develops directly within the colonic lumen and is continuously renewed through the arrival of new luminal contents from proximal segments and ongoing microbial metabolism. Importantly, the proposed lipid remodeling and nitrogen-containing exposure need not be strictly separated in time; they may partially overlap and modify each other’s effects.

The relationship between nitrogen metabolism and colorectal epithelial injury is not a new concept. Historically, the potential role of intraluminal ammonia in colorectal carcinogenesis was discussed in early studies linking its accumulation to mucosal injury and increased cellular proliferation [19]. This concept was subsequently incorporated into a broader framework of a persistently unfavorable intraluminal environment characterized by increased free ammonia, altered pH, and reduced availability of short-chain fatty acids [36]. Thus, the potential involvement of ammonia and other products of nitrogen metabolism in CRC has an established experimental and conceptual background.

The proposed model does not replace this hypothesis but places nitrogen-containing exposure within the context of an altered tissue state. The key distinction concerns the condition of the mucosa exposed to these compounds. Intraluminal ammonia is generally considered primarily as an independent injurious or promoting factor [19,36]. We propose that its biological effects may depend on the state of the epithelium and the local tissue microenvironment. If biliary–lipid loading has already been accompanied by alterations in lipid homeostasis, barrier function, and tissue responses, exposure to nitrogen-containing metabolites occurs within a biological system that differs from initially intact mucosa. This interaction represents a hypothesis of the present model and currently lacks direct experimental confirmation.

This exposure arises from the ongoing microbial metabolism of intestinal contents. As luminal contents progress through the intestine, their composition does not remain fixed but continues to change. Proteins, amino acids, urea, and other available nitrogen-containing compounds can serve as substrates for bacterial transformations, generating ammonia and a broad spectrum of other metabolites [37,38]. Slower transit is therefore relevant not only because it mechanically prolongs mucosal contact with already formed luminal contents [6,37]. It also extends the period during which the chemical environment itself continues to evolve directly at the site of epithelial exposure. Gas composition, acid–base conditions, substrate availability, and the rates of chemical and microbial reactions change simultaneously. These processes may be particularly pronounced within the near-mucosal layer, where diffusion of metabolites from the lumen intersects with microbial activity and epithelial secretion and absorption [31,32]. The resulting local chemical “cocktail” is therefore no longer simply the sum of the original dietary components, but a newly formed environment whose composition changes continuously over time.

The ammonia–ammonium system is of particular importance. In an aqueous environment, ammonia exists in dynamic equilibrium with the ammonium ion, and the relative abundance of these forms is determined primarily by pH. As pH increases, the proportion of the non-ionized form increases, enhancing its ability to cross biological membranes [19,36]. The biological effects of nitrogen loading therefore cannot be adequately assessed solely on the basis of total ammoniacal nitrogen concentration [19,36]. The same total pool may produce different levels of tissue exposure under different pH conditions.

At the same time, reducing the entire proposed nitrogen-containing load to ammonia would be an oversimplification. The colonic microbiota generates a broad spectrum of products of proteolytic fermentation, the composition of which depends on substrate availability, microbial community structure, environmental acidity, and transit time [37,38]. Ammonia/ammonium should be considered separately from other products of proteolytic metabolism: its tissue availability is governed by the specific pH-dependent NH_3_/NH_4_^+^ equilibrium, whereas other compounds have distinct chemical properties, transport pathways, and biological effects. The term “nitrogen-containing metabolic environment” is therefore used below only as a descriptive designation for the aggregate luminal exposure and does not imply a single mechanism of action. Potential additive or synergistic effects among individual metabolites likewise require independent experimental evaluation.

This is where an important link between the lipid and nitrogen-containing components of the model emerges. These exposures need not be independent. Altered biliary composition and incomplete lipid processing can modify the substrate composition of the luminal contents and microbial ecology, whereas impaired motility prolongs the time available for their further transformation [6,10,11,14,30]. When environmental composition deviates sufficiently, new combinations of chemical reactions may also emerge that are quantitatively or qualitatively atypical of the normal digestive cycle. Under these conditions, the pathological process involves not merely the accumulation of individual “waste products” but a reorganization of the entire network of transformations: which substances are generated, in what sequence, at what rate, and how rapidly they are removed or absorbed all change. At the same time, lipid remodeling of the mucosa may alter epithelial barrier properties and tissue resistance to subsequent chemical exposure. Thus, one component of the process modifies the luminal contents, another modifies the tissue with which those contents interact, and their prolonged coexistence creates conditions for mutual amplification. Lipid remodeling and nitrogen-containing exposure may not only occur sequentially but may also coexist or overlap in time. Moreover, their relative contributions may differ among regions of the same colon: alterations in tissue lipid homeostasis may predominate in some areas, features of luminal metabolic exposure in others, and their combination in still others.

Within such a system, sterile inflammation is more appropriately considered not as an independent initiating event in carcinogenesis but as a potential intermediate response to disrupted tissue homeostasis. Lipid overload, cell membrane injury, impaired barrier function, and exposure to intraluminal metabolites may sustain local damage signals even in the absence of a primary infectious inflammatory process. Monocytes and macrophages may potentially participate in this interaction, linking changes in the lipid environment to inflammatory signaling and epithelial barrier status. Thus, within the model, nitrogen-containing exposure is considered against the background of a potentially altered tissue microenvironment rather than in isolation. Initially, such a response is directed toward restoring the barrier and removing damaged cells. However, if the underlying exposure persists, repair occurs within the same environment that produced the injury. Potential clinical and endoscopic correlates of this process are nonspecific and have not yet been established; mucus production, changes in bowel habits, microscopic bleeding, erosions, edema, or heterogeneous mucosal coloration should therefore be considered only candidate variables for future studies and evaluated together with objective metabolic and morphological tissue characteristics.

The recurrence of this cycle may be more important than the intensity of any single exposure. The colonic epithelium physiologically undergoes rapid renewal; therefore, proliferation itself is not pathological [21]. A fundamentally different situation arises when successive generations of cells repeatedly develop under conditions of persistent metabolic stress. Each cycle of injury and regeneration increases the number of cell divisions during which molecular alterations may arise, while the chronic environment simultaneously determines the conditions under which newly generated cells must survive.

This leads to one of the central propositions of the present concept. At this stage, mutagenesis must be clearly distinguished from adaptation. Mutations do not arise because a cell “needs” to adapt to its chemical environment. Chronic injury, oxidative stress, and increased replicative burden may increase the probability of genetic and epigenetic alterations, but these alterations are not directionally generated. The adaptive component emerges later, at the level of selection. If a particular cellular clone randomly acquires properties that allow it to persist, recover, or proliferate more effectively in the prevailing environment, that environment may confer a selective advantage on this clone.

Accordingly, the “evolutionary” perspective proposed here does not require viewing the tumor as a programmed response of the organism. What is evolutionary is not the occurrence of a particular mutation itself, but the successive selection of cellular phenotypes within an environment that repeatedly imposes similar demands on the tissue. An alteration that might be neutral or disadvantageous under physiological conditions may confer a survival advantage under persistent metabolic instability. Clonal expansion may then become the morphologically visible outcome of a prolonged selection process that began well before the appearance of the tumor.

This interpretation brings the proposed model into closer alignment with the concept of field cancerization. Morphologically non-neoplastic mucosa is no longer regarded as a homogeneous background in which a single transformed cell arises at random. Instead, it may represent a spatially heterogeneous field of chronic metabolic stress, within which different tissue regions are at different stages of adaptation, injury, and clonal selection. Established molecular and epigenetic alterations in apparently normal mucosa in CRC demonstrate the biological possibility of such a field. Our hypothesis adds a potential metabolic dimension: local heterogeneity in lipid and nitrogen-containing exposure.

Spatial heterogeneity may be an important characteristic of such metabolic exposure, including in the distal colon. Metabolite concentrations, pH, luminal content density, and the duration of mucosal contact change during transit and may vary even within a single anatomical segment. Rather than uniform injury throughout the sigmoid colon, local regions with differing intensities of metabolic exposure may therefore be expected. In some areas, the tissue may retain compensatory capacity, whereas others may exhibit more pronounced lipid remodeling, barrier dysfunction, and regenerative activity.

This leads to one of the most important testable predictions of the present model: if the nitrogen-containing environment indeed contributes to early tissue remodeling, regions of increased nitrogen-containing exposure should show a spatial association with areas of altered mucosa or form reproducible spatial patterns relative to them. This could be tested by comparing local pH, ammonia and ammonium concentrations, the spectrum of other nitrogen-containing metabolites, and the tissue lipid profile at multiple sites within the same colonic segment. Of importance is not only the absolute concentration of a metabolite but also its spatial relationship to areas of altered mucosa and to the dynamics of the mucosa-associated environment.

The temporal sequence presents an even more complex problem. An increased concentration of nitrogen-containing metabolites in a patient with an established tumor does not demonstrate that this exposure preceded neoplasia. The tumor itself can alter motility, microbial composition, pH, surrounding tissue metabolism, and the production of nitrogen-containing compounds. Therefore, the most informative object of investigation is not the tumor tissue alone but also morphologically non-neoplastic mucosa and the local environment beyond the visible lesion.

The practical significance of this approach does not lie in using ammonia or any other individual metabolite as a ready-made biomarker of CRC. A more promising strategy may be an integrated assessment of the local environment, including transit time, pH, the nitrogen-containing metabolic profile, and the state of the adjacent mucosa. If their combination corresponds to pronounced tissue remodeling, it may characterize not the cancer itself but an intestinal region in which conditions of increased biological vulnerability have already developed.

At this stage, the potential clinical significance of the model lies primarily in defining a research strategy rather than changing diagnostic or therapeutic practice. In patients with nonspecific symptoms and morphologically non-neoplastic mucosa, a potentially informative approach may be to investigate simultaneously the characteristics of the luminal environment, the tissue lipid profile, barrier and inflammatory alterations, and dietary and metabolic factors. Only the identification of reproducible relationships among these parameters would allow their potential diagnostic, preventive, or therapeutic relevance to be evaluated subsequently.

This approach also explains why nonspecific functional symptoms should not be interpreted directly as signs of early CRC. Within the proposed model, their relevance lies not in the direct diagnosis of a tumor but in the possibility of recognizing a state in which local environmental regulation has become disrupted before the emergence of symptoms attributable to tumor growth itself.

**Figure 1:**
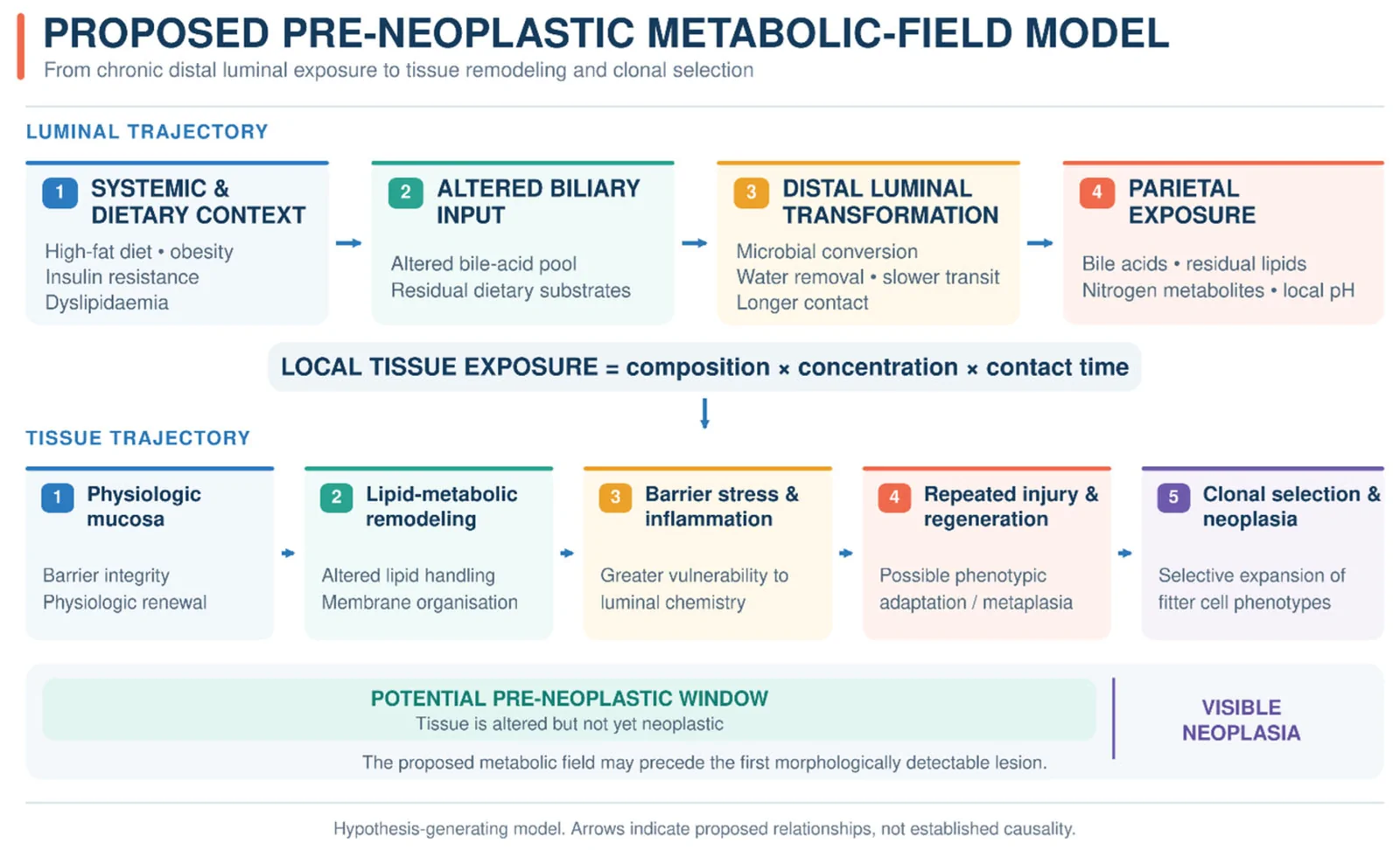
Proposed pre-neoplastic metabolic-field model of distal colorectal carcinogenesis. Systemic and dietary factors may alter biliary input and distal luminal chemistry. Microbial transformation, concentration of contents and prolonged mucosal contact may create chronic mucosa-associated exposure. In the proposed analytical sequence, lipid–metabolic remodeling of morphologically non-neoplastic mucosa is linked to barrier stress, repeated injury and regeneration, possible phenotypic adaptation, and clonal selection; these processes may partially overlap in time.

Thus, the nitrogen-containing component of the proposed concept should not be presented as a new independent carcinogenic mechanism. Its potential role is different: the biological effects of nitrogen-containing exposure may depend on the state of the tissue that develops under conditions of a chronically altered biliary–lipid and luminal environment. It is the interaction among these processes, rather than ammonia as an isolated injurious factor, that distinguishes the proposed model from earlier concepts. Within such a system, the relevant determinant is not a single molecule but the combination of a pre-existing tissue state, ongoing chemical exposure, repeated regeneration, and spatial heterogeneity of the environment. A further parameter must also be considered: its dynamic instability. Dietary intake repeatedly alters the composition of the luminal contents, after which the system attempts to return to its baseline equilibrium. However, when biliary regulation, transit, and tissue metabolism are disrupted, this return may remain incomplete. A new cycle of food intake begins before the previous state has been fully restored, leaving the tissue exposed not merely to an unfavorable but to a continuously fluctuating chemical environment. The proposed sequence is schematically illustrated in Figure 1.

The next question therefore concerns not the emergence of injury itself but its spatial consequences. If a dynamically heterogeneous metabolic field indeed develops within morphologically non-neoplastic mucosa, it is necessary to determine whether this field can influence which cellular clones acquire a selective advantage and the direction in which their subsequent expansion occurs.

## 6. From Local Clonal Selection to the Spatial Organization of Neoplastic Growth

If chronic biliary–lipid and nitrogen-containing exposures contribute to the formation of a dynamically heterogeneous metabolic field, the next question concerns not the emergence of injury itself but its spatial consequences. The emergence of a molecularly altered cellular clone does not determine either its subsequent fate or the architecture of the developing neoplasm. For progression to occur, a cell must not only survive injury but also acquire an advantage over the surrounding epithelium. Such an advantage is determined jointly by the intrinsic properties of the cell and the persistently maintained characteristics of the tissue environment in which selection occurs.

This question is particularly important in the context of the established concept of field cancerization. Studies of colorectal mucosa have shown that morphologically unaltered tissue in patients with neoplasia may harbor molecular, genetic, and epigenetic alterations, some of which vary according to their distance from the tumor [2,3,4]. Thus, the boundary between “normal mucosa” and neoplasia may be considerably less distinct than suggested by standard morphological assessment. The field itself need not be continuous or uniform. Existing evidence on field cancerization does not in itself demonstrate the existence of the metabolic field proposed here, but it establishes the biological possibility of spatial heterogeneity within morphologically non-neoplastic mucosa. If a metabolic component of such a field exists and depends on the local composition of luminal contents, characteristics of the near-mucosal layer, motility, microcirculation, and epithelial state, individual altered regions may arise focally and remain spatially separated.

The proposed model extends this concept by considering metabolic heterogeneity as one potential component of such a field. If the concentrations of lipid and nitrogen-containing metabolites, pH, the magnitude of the inflammatory response, and epithelial barrier status differ between neighboring regions of the mucosa, cells may exist under different selective conditions. The availability of structural and energy substrates may also favor cells whose metabolism is better suited to the established environment [9,20]. Thus, the same molecularly altered clone could theoretically have different biological outcomes depending on the region of the field in which it emerges.

This concept is also consistent with the established heterogeneity of CRC itself. Tumors arising in different regions of the colon differ in the prevalence of molecular pathways of carcinogenesis. Proximal tumors more commonly exhibit microsatellite instability, methylation abnormalities, and alterations associated with the serrated pathway, whereas distal tumors more frequently exhibit chromosomal instability and the classical adenoma–carcinoma sequence [5,39]. However, these differences do not form a strict boundary between two independent diseases: many molecular characteristics change gradually along the proximal–distal axis of the colon [39].

This is particularly relevant to our hypothesis. We do not propose a unique mechanism confined to the anatomical boundaries of the sigmoid colon. Rather, if the characteristics of luminal exposure indeed change as intestinal contents progress through the colon, a possible consequence may be a gradient-like rather than discrete distribution of metabolic conditions. In the present model, the sigmoid colon and rectosigmoid junction are considered potentially informative regions in which to test this hypothesis, rather than sites of established maximal metabolic exposure.

This framework raises the question of a possible relationship between the luminal–metabolic model and the established molecular heterogeneity of CRC. If the characteristics of chronic luminal exposure indeed vary along the colon, it will be necessary to determine whether these differences are associated with specific molecular pathways of carcinogenesis. In particular, a potential association between the proposed metabolically altered distal field and the adenoma–carcinoma sequence or chromosomal instability represents a testable hypothesis rather than an established causal relationship.

The spatial organization of the intestinal wall itself is equally important. The epithelium lies at the interface between two fundamentally different environments. On the apical side is the intestinal lumen, containing continuously changing luminal contents; on the basal side are the lamina propria, extracellular matrix, and microvascular and lymphatic networks. Metabolites arriving from the lumen or generated within the tissue are distributed unevenly among these compartments and are cleared at different rates. Thus, if the proposed metabolic field exists, its spatial organization should be investigated not only along the longitudinal axis of the colon but also across different compartments of the intestinal wall. As substances penetrate the mucosa, their subsequent movement is determined not only by diffusion but also by tissue uptake and drainage through blood and lymphatic capillaries, venules, and veins. This provides a physicochemical basis for spatial differences in metabolite concentration and availability but does not, in itself, indicate directional movement of neoplastic cells.

It is in this context that our hypothesis of a metabolic gradient should be considered. In its original formulation, particular emphasis was placed on a chemotactic gradient of nitrogen-containing metabolites.

At present, there is no direct evidence that the lipid or nitrogen-containing gradients proposed in this model determine the direction of CRC cell migration in vivo. Therefore, a potential relationship between the spatial distribution of metabolites and directional cell migration is considered a secondary experimental hypothesis rather than an established component of the proposed model. Testing this hypothesis will require systems in which the metabolic gradient, cellular migration, and the dependence of this response on a specific substrate are measured simultaneously [22].

Accordingly, the concept of a spatial metabolic gradient is used here in a broader sense than a simple difference in the concentration of a single compound. It refers to spatial heterogeneity in which lipid availability, nitrogen-containing compounds, pH, products of cellular and microbial metabolism, and their rates of clearance may all vary simultaneously. Such heterogeneity could potentially influence the viability and selection of cellular populations with different metabolic requirements.

Established mechanisms of tumor invasion remain the primary biological basis for the spatial spread of CRC. The classical model of endophytic growth involves acquisition of an invasive phenotype by tumor cells, degradation of the basement membrane and extracellular matrix, and the involvement of proteases, cancer-associated fibroblasts, and other components of the microenvironment [23]. Within the proposed concept, metabolic heterogeneity is considered only an additional factor that may potentially modify cell viability, proliferation, or migratory behavior within different spatial niches.

The biological plausibility of such an additional interaction is related to the substantial changes in cellular metabolic requirements following neoplastic transformation. Lipids are required for the formation of membranes and signaling structures, whereas amino acids and other available nitrogen sources contribute to nucleotide synthesis and other biosynthetic processes in rapidly proliferating cells [40]. Thus, substrates that formed part of the initial metabolic exposure may theoretically acquire a different biological role after the emergence of a neoplastic phenotype. In particular, experimental evidence indicates that exogenous fatty acids can pass from the intestinal lumen into colorectal tumors and accumulate within tumor tissue [27]. Within the proposed model, this raises the possibility that components of the environment that contributed to, or were present during, the initial injury and cellular selection may subsequently become part of the metabolic niche of the altered cell population. However, the ability of a tumor cell to utilize a particular substrate should not be equated with directional migration toward the source of that substrate.

The infiltrative pattern of tumor growth is associated with more aggressive morphological features, including tumor budding and lymphovascular invasion, and, at the molecular level, with enhanced features of epithelial–mesenchymal transition [24]. These findings support the existence of an invasive and plastic cellular phenotype but do not demonstrate that its spatial direction is determined by a metabolic gradient.

It is particularly important not to extrapolate the chemotactic hypothesis directly to lymphovascular or lymphatic spread. The vascular and lymphatic networks are indeed involved in the transport of metabolites, lipids, and cellular components, and regional lymph nodes represent metabolically active environments. However, the overlap between pathways transporting dissolved substances and tumor cells does not imply that cellular dissemination is directed by the same metabolic gradient.

From a practical perspective, this framework primarily informs the design of future studies rather than changes to current clinical management. When a polyp or early tumor is identified, testing the proposed model would require comparison of at least several regions: the neoplasm, the immediately adjacent morphologically non-neoplastic mucosa, and a distant site within the same segment. Such spatial mapping could include not only standard histology but also lipid profiling, nitrogen-containing metabolites, markers of local inflammation, and molecular features of field defects. Spatial analysis should determine whether reproducible relationships exist between the metabolic profiles of different tissue compartments and the properties of the cellular populations within them.

Of particular research value would be the detection of the proposed metabolic field in morphologically non-neoplastic tissue before neoplasia develops. Such an observation would allow direct testing of the central premise of the model that alterations in the tissue environment may precede morphologically detectable tumor growth. Longitudinal assessment of the surrounding mucosa after removal of a neoplasm would be equally important. At present, however, there is no basis for considering persistence of the proposed metabolic field an established risk factor for recurrence or metachronous neoplasia.

Thus, the formulation most relevant to the present model is that a spatially heterogeneous metabolic environment may contribute to unequal conditions for the survival and selection of cellular clones. The stronger hypothesis—that after the emergence of a corresponding metabolic phenotype, this environment can directly guide, including through chemotaxis, the spatial expansion of tumor cells—remains a separate experimental proposition. Established mechanisms of cell adhesion, proteolysis, stromal interactions, and invasion constitute the primary evidence-based mechanistic basis of tumor spread, whereas the potential influence of metabolic gradients should be investigated as an additional modifying factor.

However, once a clone capable of sustained proliferation within such a field has emerged, the relationship between the environment and the cell may change again. Lipids and nitrogen-containing compounds that may represent components of chronic metabolic exposure for normal epithelium are simultaneously potential sources of structural and energy substrates for actively proliferating tissue. A fundamentally different question therefore arises at the next stage: can the environment that contributed to the conditions selecting the neoplastic phenotype become, after its emergence, part of a metabolic niche that supports further tumor growth?

As tumor mass increases, the neoplasm itself ceases to be merely a consumer of the existing environment. A distinct cellular population emerges with its own highly active metabolism and its own spectrum of metabolic products. These compounds enter the surrounding tissue, altering local metabolic conditions and interacting with the metabolism of cells within the microenvironment [25]. Thus, the metabolic contribution of the tumor population itself is progressively superimposed on the pre-existing characteristics of the tissue environment.

**Figure 2:**
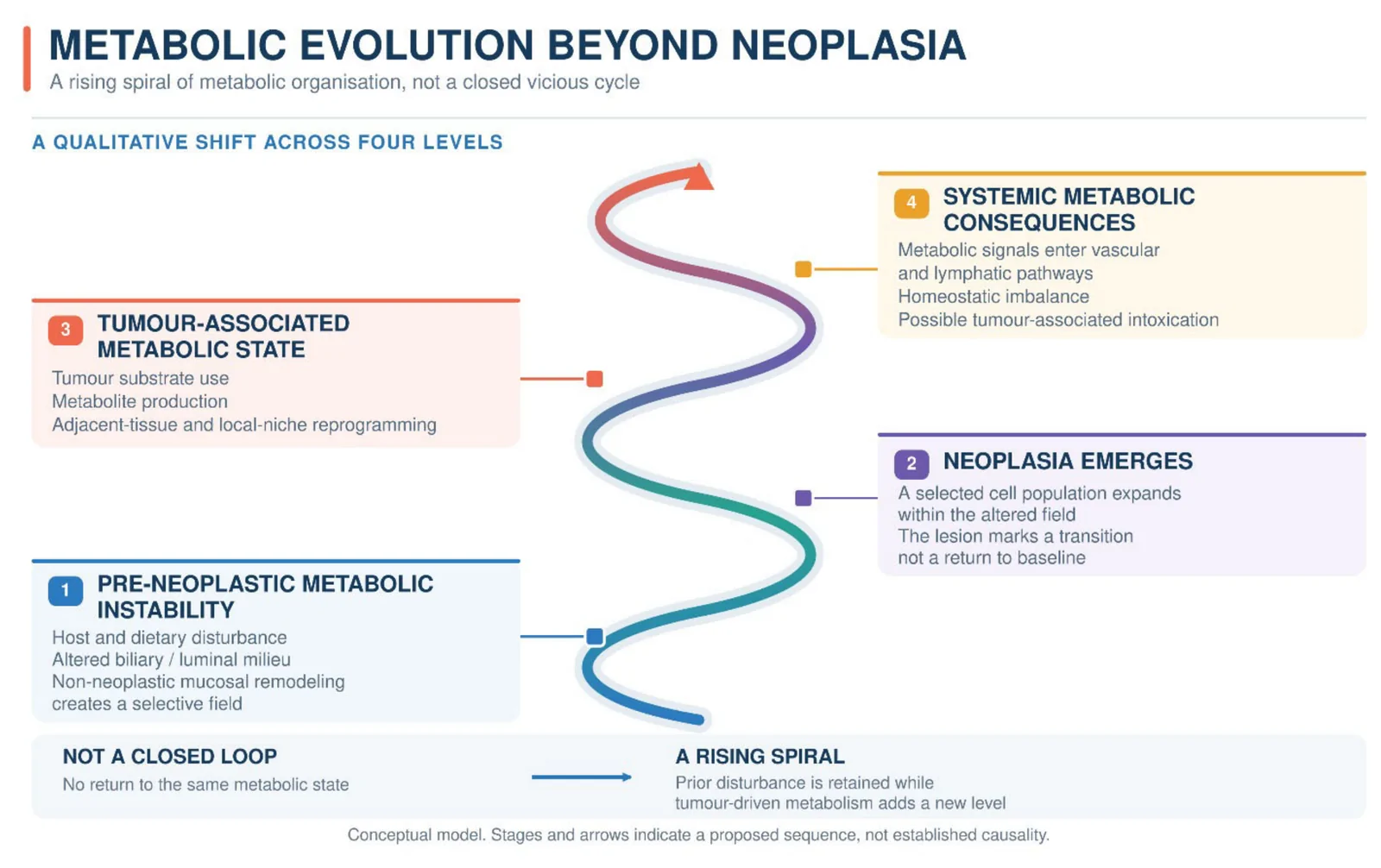
Conceptual change in the metabolic role of the tissue environment after neoplasia. Pre-neoplastic metabolic instability and putative tissue-field remodeling may contribute to conditions in which neoplastic selection occurs. Once a substantial tumor population forms, tumor-specific substrate consumption and metabolite production introduce an additional metabolic source capable of modifying the local microenvironment and potentially contributing to broader metabolic effects. The figure represents a hypothesis-generating framework rather than an established temporal or causal sequence.

At an early stage, this effect may occur locally through alterations in pH, substrate competition, intercellular signaling, and the chemical composition of the surrounding tissue, potentially creating conditions that functionally suppress neighboring normal epithelium [25]. As tumor mass increases and the vascular and lymphatic networks become involved, products of altered metabolism may extend beyond the local tissue field. With the formation of a substantial tumor cell population, a qualitatively different metabolic phenotype emerges: the original characteristics of the environment are supplemented by the tumor tissue’s own substrate consumption and metabolite production. In the present review, this transition is described as a change in the role of the tumor—from a cellular population selected within a particular environment to an independent source and modifier of the local metabolic microenvironment. This conceptual shift in the role of the tumor population is schematically illustrated in Figure 2.

## 7. Formation of Neoplasia: From an Injurious Environment to a Metabolic Niche

Up to this point, the proposed model has primarily described conditions that are unfavorable to normal epithelium. However, once a persistently proliferating cellular clone emerges, the nature of the interaction between the tissue and its environment may fundamentally change. Substrates whose excess imposed a metabolic burden on normal epithelium may, after neoplastic transformation, be incorporated into a differently organized cellular metabolism with altered biosynthetic and energetic requirements.

This concept should be considered in the context of the well-established metabolic reprogramming of tumor cells. Proliferation requires intensive synthesis of nucleotides, proteins, and membranes and, consequently, a continuous supply of carbon, nitrogen, and lipid components. Tumor cells can alter their utilization of glucose, amino acids, and fatty acids, activate lipid synthesis, remodel cholesterol metabolism, and adapt pathways of nitrogen acquisition to substrate availability [9,25,40]. Thus, a local environment rich in lipid and nitrogen-containing compounds may acquire a different biological significance once an appropriate cellular phenotype has emerged. This transition—from an injurious exposure to a potentially supportive substrate environment—is the central question addressed in this section. However, the established ability of tumor cells to reprogram their metabolism does not demonstrate that a specific pre-existing luminal environment becomes their metabolic niche; this relationship represents a separate testable component of the model.

At this point, the proposed model intersects with systemic metabolic pathways linking diet and metabolic disturbances to CRC. Obesity, insulin resistance, dyslipidemia, and alterations in insulin-like growth factor signaling may create a systemic environment that favors proliferation, while a high-fat diet may simultaneously alter biliary secretion, bile acid pool composition, and the amount of substrate entering the intestine [7,11,14]. Systemic and intraluminal mechanisms should therefore be regarded as interconnected but non-equivalent levels of exposure. The systemic environment determines the metabolic status of the tissue from the vascular side, whereas the luminal environment determines the nature of exposure from the intestinal lumen. Their effects converge at the level of the intestinal wall, although the relative contribution of each to the proposed pre-neoplastic remodeling remains a separate research question.

Following the emergence of a cellular clone with altered metabolic properties, an environment that previously represented predominantly a burden to normal epithelium may potentially confer a relative advantage on cells whose metabolic phenotype is better matched to the available substrates and local conditions.

This represents a fundamental shift in the relationship between the tissue and its metabolic environment. For normal epithelium, an excess of lipids, nitrogen-containing metabolites, and biliary components primarily represents a metabolic and chemical burden. For a highly proliferative clone, the significance of the same substrates may potentially differ. Thus, a local environment that contributed to injury and remodeling of normal mucosa at earlier stages may, following the emergence of a metabolically plastic clone, become part of a niche that supports its further expansion. Here, the term “metabolic niche” does not refer to a distinct anatomical structure or an established CRC-specific phenotype, but rather to the combination of locally available substrates and physicochemical conditions that may differentially affect normal and neoplastically altered cell populations.

If acquired molecular alterations confer increased lipid uptake, resistance to oxidative stress, altered nitrogen metabolism, or the capacity to sustain biosynthesis under an atypical substrate composition, a previously unfavorable environment may confer a relative selective advantage specifically on such a phenotype. In this sense, the significance of the environment is determined not by its absolute “favorable” or “unfavorable” nature, but by the match between available substrates and the metabolic properties of a particular cellular population.

The tumor population itself does not remain metabolically homogeneous. Genetic and phenotypic instability means that subsequent generations of cells may differ in their repertoire of transport systems and enzymes, energy requirements, and tolerance of local conditions [26]. The metabolic niche is therefore not fixed: as clonal evolution proceeds, both the properties of the cellular population and the environmental conditions that confer a selective advantage may change. This creates a dynamic system of clone–environment interactions in which changes in cellular phenotypes and remodeling of the local metabolic environment are interconnected.

Following the formation of neoplasia, a fundamentally new source of metabolic heterogeneity is added to this system—the tumor population itself. Each emerging clone not only consumes substrates but also releases its own spectrum of metabolites, which may differ from those produced by normal epithelium. For the surrounding tissue, these compounds may be atypical in their concentrations, combinations, or rates of production. Consequently, as the neoplasm grows, the initial metabolic instability that presumably existed before its formation is supplemented by a new source of instability generated by the tumor population itself.

The relationship between the tumor and the immune system should be considered similarly. CRC is not completely “invisible” to the immune system; however, intratumoral heterogeneity, alterations in antigenic composition, and metabolic remodeling of the microenvironment may contribute to immune evasion. In particular, metabolic competition and acidosis can impair the effector functions of antitumor immune cells [41]. Consequently, ongoing clonal and metabolic heterogenization of the tumor may hinder the establishment of stable and equally effective immune control across different cellular subpopulations.

Lipids are of particular interest in this context. For normal epithelium, chronic lipid excess may be associated with disrupted homeostasis and lipotoxic stress. In proliferating cells, lipid metabolism assumes a central role because newly generated cells require continuous synthesis of membranes, signaling lipids, and energy reserves. Tumor cells can increase both de novo fatty acid and cholesterol synthesis and their uptake from the surrounding environment [9,27]. Moreover, experimental evidence showing the transfer of exogenous fatty acids from the intestinal lumen into colorectal tumors [27] directly demonstrates the availability of at least some luminal lipid substrates to established neoplasia. These findings strengthen the biological basis for the proposed transition, although they do not establish that a similar lipid exposure preceded tumor development or determined its initial selection.

A similar, although more complex, logic applies to nitrogen. Rapid proliferation requires a continuous supply of nitrogen for the synthesis of amino acids, purines, and pyrimidines, and tumor cells can substantially reconfigure pathways of nitrogen uptake, redistribution, and reutilization [28,40]. In several tumor models, ammonia, traditionally regarded primarily as a toxic product of nitrogen metabolism, has been shown to re-enter metabolic reactions and serve as a nitrogen source for biosynthetic processes [28,29]. This indicates a dual, context-dependent role of ammonia in tumor metabolism: its accumulation may exert toxic effects, whereas certain tumor cells can reutilize the resulting nitrogen in anabolic reactions. However, these findings cannot automatically be extrapolated to CRC, and still less to ammonia generated or accumulated within the colonic lumen. Within the proposed model, the possibility that luminal ammonia, or nitrogen derived from it, is incorporated into the metabolism of neoplastic cells should therefore be considered a separate experimental hypothesis.

The lipid and nitrogen components of the proposed niche therefore differ in the degree of direct empirical support. For lipids, experimental evidence demonstrates that exogenous fatty acids can enter tumor tissue from the intestinal lumen [27]. For the nitrogen-containing component, the metabolic plasticity of tumor cells and the capacity for ammonia reutilization have been demonstrated in selected tumor models [28,29,40], but the sequence “luminal ammonia/ammonium → tumor-accessible nitrogen → selective advantage in CRC” has not been directly established. This distinction is fundamental to the proposed model and defines different experimental objectives for the two components.

There are therefore multiple intermediate metabolic steps between the luminal nitrogen-containing pool and the biosynthetic requirements of a tumor cell. For the present concept, the key question is not whether “cancer feeds on ammonia”, but whether a chronically altered nitrogen-containing environment can contribute to the selection of cellular phenotypes with a different organization of nitrogen metabolism and whether, after neoplasia has formed, part of the available nitrogen can enter its metabolic pathways. These two questions should be investigated separately: the first concerns selection, whereas the second concerns the metabolism of an established tumor.

This hypothesis complements rather than alters the conventional causal sequence. Tumor metabolic reprogramming is generally regarded primarily as a consequence of oncogenic alterations: mutations modify signaling pathways, after which cellular metabolism is reorganized to meet the requirements of growth. The proposed model adds the selective influence of the microenvironment to this sequence. Molecular alterations remain necessary for the neoplastic phenotype, but local substrate availability may determine which emerging metabolic phenotypes acquire a selective advantage under specific tissue conditions. The genome defines the range of possible cellular strategies, whereas the tissue environment contributes to selecting those that are most successful within a particular intestinal region.

Following the formation of neoplasia, this relationship may develop into a bidirectional interaction. Tumor growth disrupts the architecture of the mucosa and intestinal wall and can alter local motility, barrier function, inflammatory responses, and interactions with the microbiota. These changes, in turn, may further modify the chemical composition and residence time of the intestinal contents. Thus, an environment that may have preceded neoplasia does not remain unchanged after the tumor emerges; the tumor itself begins to actively modify its surroundings.

This may represent one mechanism through which local conditions for the surrounding normal tissue are altered. Competition for substrates, changes in acidity, metabolite accumulation, and immune-inflammatory remodeling can impair the function of normal and antitumor cell populations while simultaneously conferring selective advantages on tumor phenotypes adapted to such a microenvironment [25,41].

For this reason, studies of patients with established CRC cannot, by themselves, determine the original sequence of events. The detection of lipid accumulation, altered bile acids, or changes in nitrogen metabolism adjacent to a tumor is compatible with at least two opposing explanations: these alterations may have contributed to the development of neoplasia, or they may have arisen secondarily as a consequence of its presence. Reverse causality is particularly important here because the tumor is a powerful metabolic and structural modifier of the surrounding tissue. Evidence derived from tumor and peritumoral tissues should therefore be interpreted separately from evidence supporting the existence of a preceding metabolic field.

This consideration also defines the most important testable prediction of this section. If a pathological metabolic niche—or its proposed precursor, a metabolically remodeled tissue field—indeed precedes neoplasia, its principal features should be detectable not only within and immediately adjacent to the tumor but also in morphologically non-neoplastic mucosa independently of the direct influence of an established tumor. The strongest evidence would come from longitudinal data demonstrating such a phenotype before visible neoplasia develops and showing an association with its subsequent emergence. Failure to establish this temporal sequence would substantially weaken the proposed model and would instead support the interpretation that the observed metabolic alterations are predominantly consequences of tumor growth.

At present, the research perspective should not focus on measuring yet another metabolic parameter in patients with established CRC. A more informative objective is to identify and validate the proposed metabolic phenotype of at-risk tissue. This phenotype could potentially include characteristics of the bile acid pool, local lipid distribution, markers of nitrogen metabolism, pH, epithelial barrier status, and features of field defects. None of these parameters is likely to be specific in isolation. The testable hypothesis is that their spatially organized combination may characterize a tissue state preceding morphologically detectable neoplasia. Until prospectively validated, such a profile should not be regarded as a clinical risk biomarker.

Thus, the final stage of the proposed model is not simply the assertion that the tumor “feeds on” products of a preceding metabolic disturbance. Rather, it reflects a broader hypothesis: the local environment may have different biological significance for normal and neoplastically altered epithelium—ranging from a source of chronic burden and selective pressure to a component of the metabolic niche of an established tumor. Thereafter, the process becomes bidirectional: the tumor is no longer merely a product of its environment but actively modifies the local microenvironment through substrate consumption, metabolite production, and interactions with immune and stromal cells [25,41]. The central hypothesis is not the existence of these well-established tumor-associated processes, but their potential continuity with the metabolic state of morphologically non-neoplastic tissue that existed before neoplasia developed.

Nevertheless, the period preceding visible neoplasia remains the most important component of the model. At this stage, it is still possible to distinguish primary metabolic tissue remodeling from the changes subsequently generated by the tumor itself. Once a neoplastic population has formed, the system becomes considerably more complex: the pre-existing microenvironment continues to participate in cellular selection, while the tumor simultaneously remodels this microenvironment and creates new selective conditions. The priority for testing the model is therefore not further characterization of the already extensively studied metabolism of established CRC, but reconstruction of the metabolic state of morphologically non-neoplastic tissue before tumor development. Establishing or refuting such a preceding state will determine whether the proposed link among biliary–luminal exposure, tissue remodeling, and the subsequent tumor metabolic niche represents a genuine biological sequence.

## 8. Discussion

One of the distinctive features of the present review is its interdisciplinary nature. A substantial proportion of the evidence required to construct the proposed model has emerged not directly from surgical or clinical oncology but from adjacent fields, including biochemistry, cell biology, digestive physiology, histology, microbiology, immunology, metabolomics, and bioengineering. Integrating these otherwise separate levels of evidence makes it possible to address a question that is difficult to formulate within any single discipline: does the visible tumor represent the beginning of the pathological process, or merely its late morphological manifestation? This perspective may be particularly relevant to CRC because dietary exposure, biliary processing, the state of the luminal contents, intestinal wall responses, clonal evolution, and tumor growth itself may involve prolonged and partially overlapping interactions that have traditionally been investigated by different disciplines.

Contemporary colorectal cancer prevention is largely centered on the timely detection of an already established morphological substrate: an adenoma, serrated lesion, dysplasia, or early carcinoma. This approach has demonstrated its effectiveness and remains the foundation of screening [42]. However, the proposed model raises an additional research question: before the first endoscopically visible lesion appears, are there local tissue alterations that can be objectively characterized? If, in at least some cases, neoplasia is preceded by prolonged alterations in the local metabolic environment and a persistent mucosal response, relevant biological information may emerge before morphologically detectable neoplasia.

This represents the principal practical and research significance of the proposed concept. It does not imply replacement of existing screening strategies, nor does it suggest that nonspecific mucosal alterations should be interpreted as precancerous lesions. Rather, it potentially broadens the object of clinical and research observation: alongside the pathological lesion itself, the state of the tissue in which it develops may become an independent object of investigation. Morphologically non-neoplastic mucosa should therefore not be assumed a priori to represent biologically intact tissue.

This approach has an empirical basis in the concept of field cancerization. Molecular and epigenetic alterations can be detected in macroscopically normal mucosa beyond the tumor, and in some studies their magnitude varies with distance from the neoplasm [2,3,4]. The proposed model adds a testable metabolic component to this concept. Before morphologically evident neoplasia develops, there may hypothetically exist not only a molecularly altered cellular field but also a metabolically altered tissue field characterized by changes in lipid metabolism, interactions with biliary components, the local chemical environment, and the functional state of the intestinal wall. The existence of such a preceding metabolic field, as distinct from the broader phenomenon of field cancerization, requires independent validation.

From an endoscopic perspective, this concept provides a rationale for investigating the mucosa surrounding an identified lesion. When a polyp is detected, its size, morphology, surface characteristics, vascular pattern, and signs of possible invasion are routinely assessed. Following removal, attention shifts primarily to the histological findings and subsequent endoscopic surveillance. Within the proposed model, characterization of the lesion itself is supplemented by another question: are there reproducible alterations in the surrounding morphologically non-neoplastic tissue that are associated with the location of the neoplasm and the proposed tissue field?

In this regard, the surrounding mucosa may potentially contain information that cannot be obtained by examining the polyp alone. Local flattening or remodeling of the mucosal relief, edema, changes in mucus coating, signs of chronic injury, and motility characteristics of the corresponding segment are nonspecific in themselves and should not be interpreted as signs of a precancerous state. Nor has it been established that their combination constitutes a distinct clinical or endoscopic phenotype. Such characteristics are therefore of interest primarily as predefined variables for prospective studies and should be evaluated in relation to the tissue lipid profile, barrier and inflammatory alterations, the local chemical environment, and subsequent risk of neoplasia.

**Figure 3:**
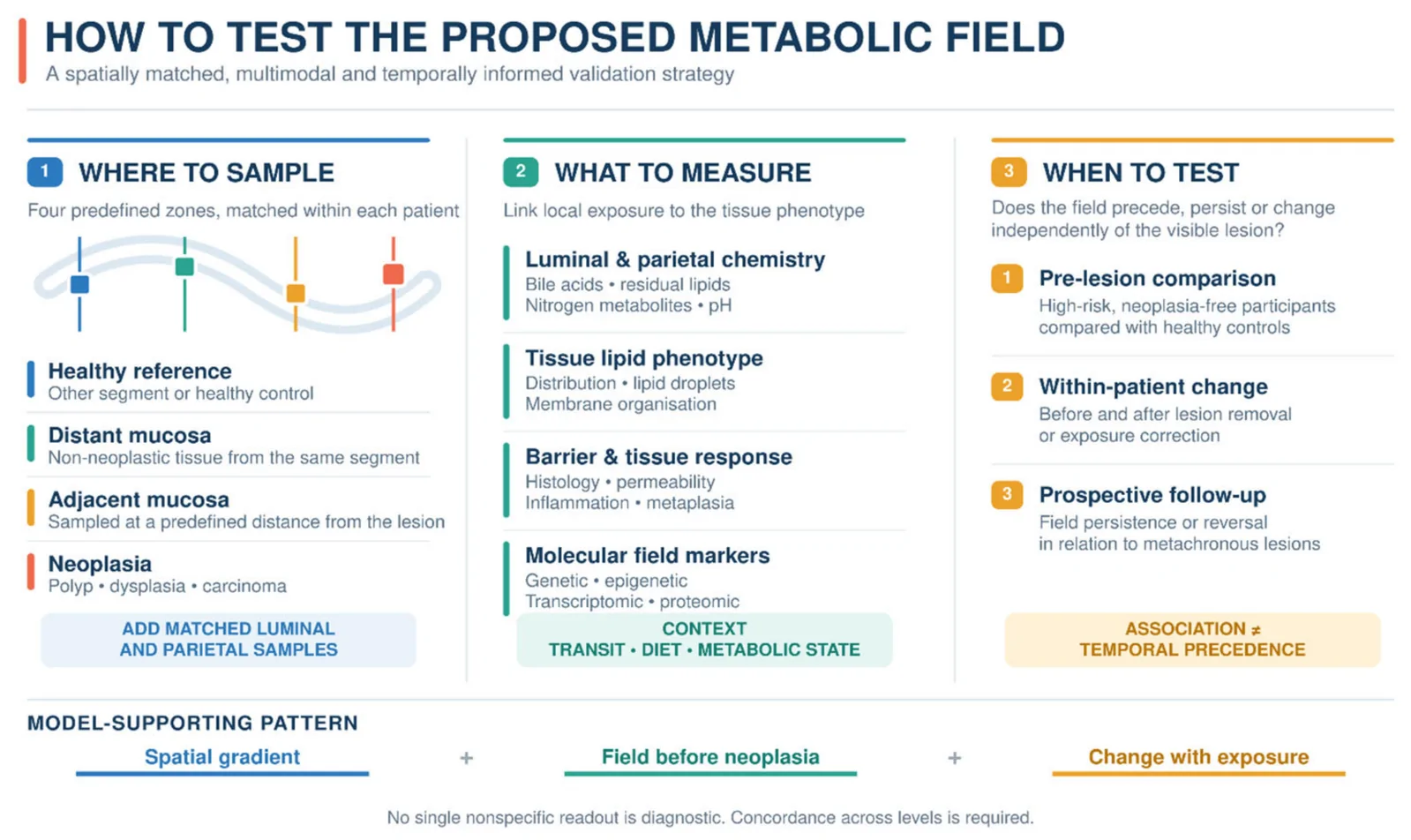
Spatial and temporal strategy for testing the proposed metabolic field. The lesion, adjacent mucosa, distant mucosa from the same segment, and a healthy reference should be sampled using predefined distances and integrated with luminal and mucosa-associated chemistry, tissue lipid phenotype, barrier and inflammatory responses, and molecular field markers. Cross-sectional mapping should be complemented by neoplasia-free high-risk cohorts, within-patient comparisons, and prospective follow-up. Support requires concordance across levels rather than a single nonspecific marker.

This perspective suggests a distinct research-oriented biopsy strategy. The informative target would include not only the lesion itself but also adjacent and more distant morphologically non-neoplastic mucosa. The distance from the neoplasm may be particularly important. Biopsy specimens obtained immediately adjacent to the lesion, from a more distant site within the same segment, and from morphologically intact mucosa in another colonic region would allow investigation of the spatial gradient of the proposed alterations but should not be interpreted a priori as sequential stages of a single process. If a metabolic field does exist, the boundary between “tumor” and “normal tissue” may prove substantially less distinct at the biochemical level than on standard endoscopic assessment. The proposed strategy for spatial and temporal testing of the model is presented in Figure 3.

Metaplastic remodeling of the epithelium warrants separate consideration within this model. Chronic injury and inflammation may be accompanied by adaptive remodeling of the epithelial phenotype, including the development of metaplastic changes in certain pathological conditions of the intestine [43]. Within the proposed model, this raises the question of whether a similar principle of adaptive reprogramming may also be relevant during prolonged metabolic exposure. At present, this hypothesis does not justify considering metaplasia an obligatory or specific intermediate step in sporadic colorectal carcinogenesis. From a research perspective, the relevant question is therefore not merely whether metaplastic alterations are present as an isolated histological finding, but whether they show a spatial relationship with regions of chronic injury and characteristics of the surrounding environment. Until such evidence becomes available, metaplastic adaptation should be regarded as a possible, but not obligatory, tissue response.

Particular attention in the proposed model is given to the sigmoid colon and rectosigmoid junction. This does not imply that these regions are regarded as the exclusive sites at which a metabolic mechanism of CRC operates. However, features of distal transit, prolonged residence of intestinal contents in some individuals, and ongoing microbial fermentation make the sigmoid colon and rectosigmoid junction reasonable candidate sites for spatial testing of the model [6]. Moreover, established differences between proximal and distal tumors demonstrate that CRC cannot be regarded as a biologically homogeneous disease irrespective of anatomical location [5,39]. The proposed model adds another level for investigation alongside molecular heterogeneity: differences in luminal–tissue interactions along the colon. However, the assumption that the proposed field is maximal or preferentially expressed in the sigmoid colon should not be regarded as established and requires direct intersegmental comparison.

From a clinical perspective, this issue is particularly relevant in patients whose endoscopic findings are accompanied by functional disturbances. Slower transit, a tendency toward retention of intestinal contents, bloating, altered bowel habits, and episodic discomfort are extremely common and, in the overwhelming majority of cases, do not indicate neoplasia. Their use as independent markers of CRC would therefore be inappropriate. Their relevance within the proposed model is limited to investigating the relationship between motility and the duration of local chemical exposure. Slower transit may prolong mucosal contact with intestinal contents and alter the pattern of microbial fermentation [6]. Within the proposed model, the relevant question is how this temporal component interacts with the chemical composition of the luminal and mucosa-associated environments.

The biliary component should be considered similarly. Evidence linking secondary bile acids to CRC extends beyond the view of bile solely as a physiological component of digestion [8,11,12,13,14]. Alterations in bile acid pool composition and microbial transformation modify the chemical environment to which the colon is exposed. In this context, high-fat diets, obesity, insulin resistance, and dyslipidemia are relevant both as systemic risk factors and as factors capable of modifying individual components of biliary–intestinal exposure [7,11,14]. This provides a biological basis for investigating interactions between systemic metabolic status and local processes within the intestinal wall without assuming direct causal equivalence between them.

For clinical studies, this relationship suggests that a patient’s metabolic status should be considered when characterizing the local tissue and luminal phenotype rather than treated exclusively as a background comorbidity. However, current evidence is insufficient to incorporate the proposed metabolic field into existing models of individual CRC risk stratification. Such an application should be considered only after the independent prognostic value of the relevant parameters has been established.

Patients who have undergone removal of a localized lesion are particularly interesting from this perspective. Following removal of colorectal neoplasia, the risk of subsequent metachronous lesions remains heterogeneous and depends, among other factors, on the characteristics of the index lesions, which form the basis of current post-polypectomy surveillance stratification [44]. The proposed model raises an additional question: could part of this risk be associated not only with the characteristics of the removed lesion but also with persistence of a biologically altered tissue field? If the surrounding tissue indeed retains the proposed metabolic phenotype and remains exposed to a similar luminal environment, a testable hypothesis arises as to whether persistence of such a field may contribute to the risk of subsequent metachronous lesions.

This does not require any change to the existing logic of clinical surveillance. On the contrary, post-polypectomy cohorts provide a useful setting in which to test the hypothesis: it can be determined whether the proposed tissue and metabolic characteristics persist after lesion removal and whether they provide prognostic information beyond currently used morphological risk factors. Only if such an independent association is confirmed could the state of the surrounding tissue be considered a potential additional level of risk stratification.

Notably, emerging evidence already points to the potential clinical relevance of characteristics of non-neoplastic tissue. In a recent study, the molecular and microbiological phenotype of non-tumor-bearing colorectal tissue distant from the tumor identified a microenvironment associated with poorer disease-free and overall survival [45]. These observations do not establish a causal role for a pre-existing metabolic field because the tissue was examined in patients with established CRC; nevertheless, they support the broader proposition that the biological state of non-neoplastic mucosa may contain prognostic information not captured by analysis of the tumor alone. At the same time, they emphasize the problem of reverse causality: the identified phenotype may either have preceded the tumor or have developed or changed under its influence.

The same research logic applies to radical surgery for CRC. The oncological radicality of surgery is determined by established principles governing resection of the primary tumor and regional lymphatic structures and does not require the introduction of an additional clinical criterion. Nevertheless, a separate biological question can be asked regarding the extent to which resection alters the proposed metabolic field. After removal of the affected segment, it remains unclear whether the pathological environment disappears or whether only the anatomical region in which it was most pronounced has been removed. Surgical treatment of CRC can alter the intestinal microbiome and metabolome; comparisons before surgery and approximately one year afterward have demonstrated changes in microbial composition and several fecal metabolites, including DCA-producing microorganisms and features related to bile acid metabolism [46]. However, the nature of these changes after different types of resections and their relevance to the proposed metabolic field remain insufficiently characterized. The previously proposed working term “metabolic radicality” should therefore not be used as an analogue of oncological radicality; instead, this question is more appropriately framed as an investigation of the biological consequences of resection for the remaining tissue and intestinal environment.

A similar question arises in relation to neoadjuvant and adjuvant chemotherapy and molecularly targeted treatment. These approaches can reduce tumor burden, eliminate susceptible clones, and alter the composition of the residual cellular population. However, their effects on the biliary–lipid and nitrogen-containing environment considered within the model as a possible precursor of neoplasia represent a separate question. Investigating this environment before and after treatment could determine whether characteristics of the proposed field persist independently of tumor response. The relationship between such persistence, the biology of residual cellular populations, and subsequent progression could then be examined separately.

This does not establish persistence of a metabolic field as a proven cause of recurrence or metastasis. Nor does the model currently provide a basis for modifying existing anticancer treatment regimens or supplementing them with metabolically targeted therapy. Its significance lies in formulating a testable hypothesis: are characteristics of the surrounding tissue independently associated with the persistence of residual clones, recurrence, or metachronous neoplasia? If such an association is confirmed, targeting the supportive microenvironment could become a distinct direction for subsequent interventional studies.

The proposed lipid–metabolic remodeling may be particularly informative in this context. We use this term not to designate a distinct disease of the colon but to describe a potential tissue phenotype of chronic lipid dysregulation. If its existence is confirmed morphologically and biochemically, establishing its functional significance and temporal relationship to neoplasia will become essential. Lipid overload can alter intracellular lipid storage and distribution, membrane organization, and associated signaling processes [16,17,35]. Within the proposed model, the combination of these alterations with barrier dysfunction and local inflammation is considered a candidate tissue phenotype that must be distinguished both from genuinely intact mucosa and from secondary alterations caused by an already established tumor.

Despite advances in non-invasive screening methods, clinically identifiable precursor or early neoplasia is ultimately characterized by detection of an already established neoplastic process. The hypothesis of metabolically altered mucosa raises the possibility of an earlier biological state. If the corresponding phenotype indeed precedes adenoma or other forms of neoplasia, it may define a research-identifiable intermediate level between the presence of risk factors and morphologically detectable neoplasia. Such an intermediate level is currently difficult to characterize: clinicians may determine that a patient has an increased risk of CRC or detect an existing lesion, but it is considerably more difficult to establish the specific region of the intestine in which that risk is beginning to manifest at the tissue level.

The proposed concept provides one possible approach to addressing this gap. Systemic factors determine susceptibility, characteristics of biliary and intestinal metabolism may contribute to local exposure, and the state of the mucosa may reflect the tissue response to that exposure. Testing the model may therefore require not an isolated laboratory or endoscopic parameter but the integration of three levels of information: the patient’s metabolic state, characteristics of the local intestinal environment, and the phenotype of the mucosa itself.

Finally, the proposed model broadens the research framework of early detection. The earliest target of investigation when testing the hypothesis need not necessarily be a cell that has already acquired a neoplastic phenotype. It may instead be a region of mucosa that does not yet contain a tumor but is characterized by a reproducible combination of alterations in the local environment and a persistent tissue response. Until a temporal relationship with subsequent neoplasia has been established, such a state should not be designated as precancerous or used for clinical diagnosis.

If the model is confirmed, an intermediate tissue state may be identified between a risk factor and neoplasia, in which persistent functional and metabolic alterations are already detectable but a neoplastic phenotype is still absent. If such a pre-neoplastic metabolic state exists and retains functional plasticity, its reversibility becomes a critical testable question. In particular, it will be necessary to determine whether modifications in dietary load, biliary dynamics, transit, or other components of the local environment can alter or normalize the corresponding tissue phenotype before a stable neoplastic population develops. Only demonstration of reversibility, temporal precedence over neoplasia, and an association between intervention and subsequent risk reduction would justify considering such a state a potential target for primary prevention rather than merely an associated marker.

Thus, at the present stage, the practical significance of the proposed concept lies primarily in establishing a research program that extends beyond the question of when to detect a tumor. It requires sequential evaluation of three objects: the neoplasm itself, the tissue in which it developed, and the metabolic environment interacting with that tissue.

If the model receives experimental and prospective clinical validation, its relevance could potentially extend across several stages of the clinical trajectory—from investigation and subsequent potential stratification of tissue-level risk to post-polypectomy and postoperative surveillance and the development of interventions targeting modifiable components of the local environment. However, translation of the conceptual model into such applications requires sequential confirmation of the existence of the proposed tissue phenotype, its temporal precedence over neoplasia, its prognostic value, and, ultimately, its modifiability. The central question of the present work is therefore not whether current clinical practice should be changed, but whether persistent and potentially reversible conditions that facilitate tumor development exist before the tumor emerges and whether they persist after removal of the neoplastic lesion.

## 9. Conclusions

The proposed concept shifts attention from established neoplasia to the state of the tissue that may precede its emergence. At the center of this model is not a single carcinogenic metabolite, but a persistent biliary–metabolic environment whose interaction with intestinal transit, microbial metabolism, and tissue responses may generate sustained local exposure.

A key intermediate component of the proposed model is the hypothesized lipid–metabolic remodeling of morphologically non-neoplastic mucosa. Within this concept, it represents not a new diagnosis but a testable tissue phenotype in which chronically altered lipid exposure may be accompanied by persistent changes in local lipid homeostasis and the functional properties of the epithelium. Direct demonstration of this transition from luminal lipid loading to persistent remodeling of non-neoplastic tissue remains one of the key requirements for validation of the model. Subsequent exposure to nitrogen-containing metabolites and other components of the altered intraluminal environment would then occur against the background of an already altered tissue state. We propose that the interaction among these processes may create conditions for repeated injury, regeneration, and selection of cellular clones better able to persist within the resulting environment. Lipid and nitrogen-containing exposures need not occur in a strictly sequential manner and may partially overlap in time.

This perspective allows early CRC to be considered as a process developing not only within an individual cell but also within a progressively changing tissue field. In this sense, the proposed model extends the concept of field cancerization by introducing the hypothesis of a metabolic component: morphologically unaltered mucosa may already harbor molecular and epigenetic alterations [2,3,4], whereas the existence of persistent alterations in lipid metabolism and the local biliary–metabolic environment preceding neoplasia requires direct validation. Once a neoplastic clone emerges, the same environment may potentially change its role—from a source of chronic metabolic burden to a component of a metabolic niche in which cells with a corresponding metabolic phenotype acquire an additional advantage.

Thus, future approaches to CRC risk assessment could potentially be complemented by investigation of an earlier tissue level, provided that a reproducible metabolic phenotype preceding morphologically detectable neoplasia is demonstrated. If such a metabolically remodeled field is confirmed, the next fundamental question will concern its reversibility. It will be necessary to determine whether modification of dietary exposure, biliary dynamics, intestinal transit, or other components of the local environment can alter the tissue phenotype and whether such modification is accompanied by a subsequent reduction in neoplastic risk. Only this sequence of evidence would justify moving from the description of a potential at-risk tissue state toward considering it as a possible target for primary prevention.

We also regard this concept as an open research hypothesis. The objective is not to confirm every component at all costs, but to determine which elements of the proposed sequence are reproducible, where its boundaries lie, and which observations require revision of the original model. Independent testing of the most uncertain links is particularly important: the transition from chronic luminal lipid exposure to persistent remodeling of morphologically non-neoplastic mucosa, a possible alteration in the susceptibility of such tissue to nitrogen-containing metabolites, and the proposed relationship between spatial metabolic gradients and subsequent clonal expansion. Findings that do not support these relationships will be no less informative in defining the boundaries of the model than positive observations.

The proposed model is not intended to oppose existing molecular models of colorectal carcinogenesis or to replace established mechanisms of mutagenesis, clonal evolution, field cancerization, or tumor metabolic reprogramming. Its purpose is different: to test whether the state of the local tissue and luminal environment may contribute to shaping the conditions under which injury, regeneration, and subsequent selection of cellular clones occur. This perspective allows the tumor to be considered not only as the result of the sequential accumulation of cellular alterations but also as the result of their selection within a specific tissue context.

The central idea of the present article is therefore the need to investigate not only the cell that has acquired a neoplastic phenotype, but also the tissue environment in which its injury, adaptation, and clonal selection occurred. If specific characteristics of this environment are shown to consistently precede neoplasia, confer selective advantages on emerging cellular phenotypes, and persist after tumor development or removal, this would make it possible to integrate the metabolic history of non-neoplastic tissue with the subsequent evolution of CRC within a single experimentally testable model.

## Figures and Tables

**Table 1:** Summary of the evidence base supporting individual components of the proposed model. Author–year citations in this table identify the studies underlying the evidence classification and correspond to references cited numerically in the main text. The categories indicate the degree of direct empirical support for individual components of the proposed model and do not constitute a formal evidence-grading system.

Component of the Model	Human Evidence	Experimental Evidence	Indirect Support	Status Within the Proposed Model
**Altered biliary–metabolic exposure and effects of secondary bile acids**	Associations between metabolic factors and CRC; data on bile acid pool composition (Han et al., 2021 [7]; Chen et al., 2020 [10])	Epithelial injury, oxidative stress, and carcinogenesis-associated effects of secondary bile acids (Liu et al., 2022 [8]; Yang et al., 2022 [11]; Yao et al., 2022 [12]; Cong et al., 2024 [13])	Physiology of enterohepatic circulation and microbial transformation of bile acids (Ocvirk and O’Keefe, 2021 [14]; Di Ciaula et al., 2017 [15])	Biologically plausible component; individual mechanisms are supported, whereas their integration into a unified chronic exposure remains conceptual
**Lipid–metabolic remodeling of morphologically non-neoplastic mucosa**	Direct evidence of persistent pre-neoplastic lipid remodeling is limited; alterations in morphologically non-neoplastic mucosa in CRC have been described (Dampier et al., 2020 [2]; Ghosh et al., 2022 [3]; Ikuta et al., 2024 [4])	Evidence supports effects of lipid loading, lipid droplets, and alterations in membrane organization (Mollinedo and Gajate, 2020 [16]; Olzmann and Carvalho, 2019 [17])	Mechanisms of lipid adaptation in other tissues, including the liver (Xu et al., 2022 [18])	Central testable hypothesis; direct evidence for the proposed phenotype is currently lacking
**Lipid remodeling → altered susceptibility to ammonia/other products of proteolytic fermentation**	No direct evidence	Evidence for effects of ammonia on the intestinal epithelium (Visek, 1978 [19])	Indirect mechanistic support only	Hypothetical link
**Repeated injury and regeneration → conditions for clonal selection**	Field cancerization and alterations in morphologically non-neoplastic mucosa have been demonstrated in patients with CRC (Dampier et al., 2020 [2]; Ghosh et al., 2022 [3]; Ikuta et al., 2024 [4])	Experimental evidence supports a role for chronic injury and regeneration	General mechanisms of regenerative plasticity and clonal evolution (Rane et al., 2023 [20]; Sphyris et al., 2021 [21])	Biologically plausible integration; the proposed causal and temporal sequence has not been established
**Spatial metabolic gradients → directional clonal expansion**	No direct evidence	Limited support from experimental models of gradient-dependent tumor cell behavior (Ascione et al., 2024 [22])	General evidence on tumor mechanobiology and invasive growth patterns (Brás et al., 2022 [23]; Kehusmaa et al., 2026 [24])	Secondary hypothesis requiring direct testing in CRC
**Metabolic niche after the emergence of neoplasia**	Metabolic reprogramming and heterogeneity of CRC are established (Salita et al., 2022 [9]; Zhong et al., 2022 [25]; Hirose and Taniguchi, 2023 [26])	Experimental evidence demonstrates utilization of different metabolic substrates by tumor cells (Janssen et al., 2026 [27]; Kurmi and Haigis, 2020 [28]; Chen et al., 2025 [29])	General principles of tumor and immune metabolism	Supported conceptual integration; the proposed “change in role” of the pre-existing environment remains hypothetical

## Data Availability

No new data were created or analyzed in this study. Data sharing is not applicable to this article.

## Supplementary Materials

The following supporting information can be downloaded at: https://www.mdpi.com/article/10.3390/cancers18193160/s1, Table S1: Literature search strategy used for the narrative review and conceptual synthesis.
