# Supplementary material for: From Bile Dysmetabolism to Lipid–Metabolic Remodeling of the Distal Colon: A Vectorial Concept of Colorectal Carcinogenesis (Part I)
Source: Cancers (Basel). 2026 Sep 30;18(19):3160. doi: 10.3390/cancers18193160 (PMC13664052; doi:10.3390/cancers18193160)
Supplement: Supplementary file 1 [file cancers-18-03160-s001.zip › cancers-4580874-supplementary.pdf]

Supplementary Table S1. Literature search strategy used for the narrative review and conceptual synthesis

Search domain	Representative search terms and combinations	Main purpose within the review
**Bile acid metabolism and enterohepatic circulation**	(“bile acid” OR “biliary metabolism” OR “enterohepatic circulation” OR “bile acid pool”) AND (“colon” OR “colorectal” OR “intestinal”)	To identify evidence on bile acid physiology, enterohepatic circulation, determinants of bile acid composition, and their relevance to the colonic luminal environment.
**Secondary bile acids and colorectal carcinogenesis**	(“secondary bile acid” OR “deoxycholic acid” OR “lithocholic acid”) AND (“colorectal cancer” OR “colorectal carcinogenesis” OR “colon tumorigenesis”)	To evaluate experimental and human evidence linking secondary bile acids with epithelial injury, oxidative stress, DNA damage, inflammation, proliferation, and colorectal tumorigenesis.
**Dietary lipids and intestinal/colonic lipid metabolism**	(“dietary fat” OR “dietary lipid” OR “intestinal lipid metabolism” OR “colonic lipid metabolism” OR “lipid absorption”) AND (“colon” OR “colonic mucosa” OR “colorectal”)	To characterize intestinal lipid handling and assess whether dietary or luminal lipid exposure may influence the metabolic state of colonic tissue.
**Tissue lipid remodeling and lipid droplets**	(“lipid remodeling” OR “lipid metabolic reprogramming” OR “lipid droplet” OR “intracellular lipid” OR “membrane lipid”) AND (“colon” OR “colonic mucosa” OR “colorectal epithelium” OR “colorectal cancer”)	To distinguish luminal lipid exposure, epithelial lipid uptake, intracellular lipid storage, membrane changes, and tissue-level lipid–metabolic remodeling.
**Morphologically non-neoplastic mucosa and field cancerization**	(“normal adjacent mucosa” OR “non-tumor mucosa” OR “normal-appearing mucosa” OR “histologically normal mucosa” OR “field cancerization” OR “field effect” OR “field defect”) AND (“colorectal cancer” OR “colorectal neoplasia”)	To identify molecular, genetic, epigenetic, transcriptomic, and metabolic alterations in morphologically non-neoplastic colorectal mucosa and assess their spatial relationship to neoplasia.
**Colonic transit and regional luminal exposure**	(“colonic transit” OR “intestinal transit” OR “slow transit” OR “transit time” OR “mucosal contact time”) AND (“colon” OR “distal colon” OR “sigmoid colon” OR “colorectal”)	To evaluate whether regional differences in transit and contact time could contribute to spatial heterogeneity of luminal and parietal exposure.
**Microbial fermentation and luminal metabolism**	(“gut microbiota” OR “intestinal microbiota” OR “microbial metabolism” OR “microbial fermentation”) AND (“colon” OR	To characterize microbial transformation of luminal substrates and its potential contribution to the local metabolic environment.

Search domain	Representative search terms and combinations	Main purpose within the review
	"colorectal") AND ("metabolite" OR "luminal environment")	
**Ammonia/ammonium and colorectal epithelium**	("ammonia" OR "ammonium" OR "NH3" OR "NH4") AND ("colon" OR "colonic epithelium" OR "colorectal")	To evaluate ammonia/ammonium production, pH-dependent chemical equilibrium, epithelial exposure, and reported biological effects on colorectal tissue.
**Proteolytic fermentation products**	("proteolytic fermentation" OR "protein fermentation" OR "amino acid-derived bacterial metabolites" OR "nitrogenous metabolite") AND ("colon" OR "colorectal")	To assess microbial products of protein and amino-acid metabolism separately from ammonia/ammonium and to characterize their potential epithelial effects.
**Epithelial injury, regeneration, and phenotypic plasticity**	("epithelial injury" OR "epithelial regeneration" OR "wound healing" OR "epithelial plasticity" OR "metaplasia") AND ("colon" OR "colonic epithelium" OR "colorectal")	To examine mechanisms by which repeated injury and regeneration may alter epithelial phenotype and create conditions relevant to clonal selection.
**Clonal evolution and selection**	("clonal evolution" OR "clonal selection" OR "somatic evolution" OR "premalignant clone") AND ("colon" OR "colorectal" OR "normal tissue" OR "premalignant tissue")	To integrate established concepts of clonal evolution in normal, premalignant, and neoplastic tissues with the proposed tissue-field framework.
**Spatial heterogeneity and proximal–distal differences**	("spatial heterogeneity" OR "regional heterogeneity" OR "proximal distal" OR "right-sided left-sided" OR "colorectal subsite") AND ("colorectal cancer" OR "colorectal mucosa")	To assess regional molecular and biological heterogeneity across the colorectum without presuming that the distal colon represents the site of maximal exposure.
**Metabolic gradients and spatial tumor behavior**	("metabolic gradient" OR "nutrient gradient" OR "microenvironmental gradient" OR "nutrient availability") AND ("tumor growth" OR "cancer cell migration" OR "clonal expansion" OR "tumor invasion")	To evaluate experimental evidence that spatial differences in nutrient or metabolic conditions can influence tumor-cell behavior and to assess the biological plausibility of the model's secondary spatial hypothesis.
**Colorectal tumor metabolic reprogramming**	("metabolic reprogramming" OR "tumor metabolism" OR "lipid metabolism" OR "nitrogen metabolism" OR "metabolic heterogeneity") AND ("colorectal cancer" OR "colon cancer")	To characterize metabolic adaptations occurring after neoplastic transformation and distinguish them from the proposed pre-neoplastic tissue-field changes.

Databases: PubMed/MEDLINE, Scopus, and Web of Science.
Last structured search: 1 July 2026.
Date restrictions: No lower publication-date limit was applied.
Additional identification of studies: Reference lists of relevant original studies and review articles were screened for additional publications.

Selection approach: Original experimental and clinical studies directly addressing individual components of the proposed framework were prioritized. Systematic and narrative reviews were used to characterize broader biological domains and to identify relevant primary studies. When several sources addressed the same mechanism, preference was given to more recent publications, original studies, and studies directly involving colorectal tissue or colorectal cancer. Earlier studies were retained when they represented historically important or foundational experimental observations without an equivalent contemporary primary study.

Interpretation of the search strategy: Because this study was designed as a narrative review with conceptual synthesis rather than a systematic review or meta-analysis, the search domains and representative term combinations were used iteratively and were adapted to individual mechanistic questions. They should therefore be interpreted as a transparent description of the literature-identification strategy rather than as a single fixed systematic search algorithm.
